# Phenotypic plasticity explains apparent reverse evolution of fat synthesis in parasitic wasps

**DOI:** 10.1038/s41598-021-86736-8

**Published:** 2021-04-08

**Authors:** Bertanne Visser, Hans T. Alborn, Suzon Rondeaux, Manon Haillot, Thierry Hance, Darren Rebar, Jana M. Riederer, Stefano Tiso, Timo J. B. van Eldijk, Franz J. Weissing, Caroline M. Nieberding

**Affiliations:** 1 Evolution and Ecophysiology Group, Biodiversity Research Centre, Earth and Life Institute, UCLouvain, Croix du Sud 4-5, 1348 Louvain-la-Neuve, Belgium; 2 Chemistry Research Unit, Center of Medical, Agricultural, and Veterinary Entomology, Agricultural Research Service, United States Department of Agriculture, 1600 SW 23rd Drive, Gainesville, FL 32608 USA; 3 Ecology of Interactions and Biological Control Group, Biodiversity Research Centre, Earth and Life Institute, UCLouvain, Croix du Sud 4-5, 1348 Louvain-la-Neuve, Belgium; 4 Department of Biological Sciences, Emporia State University, 1 Kellogg Circle, Campus Box 4050, Emporia, KS 66801 USA; 5 Groningen Institute of Evolutionary Life Sciences, University of Groningen, Nijenborgh 7, 9747 AG Groningen, The Netherlands; 6 Evolutionary Ecology and Genetics Group, Biodiversity Research Centre, Earth and Life Institute, UCLouvain, Croix du Sud 4-5, 1348 Louvain-la-Neuve, Belgium

**Keywords:** Evolution, Ecology, Evolutionary ecology

## Abstract

Numerous cases of evolutionary trait loss and regain have been reported over the years. Here, we argue that such reverse evolution can also become apparent when trait expression is plastic in response to the environment. We tested this idea for the loss and regain of fat synthesis in parasitic wasps. We first show experimentally that the wasp *Leptopilina heterotoma* switches lipogenesis on in a fat-poor environment, and completely off in a fat-rich environment. Plasticity suggests that this species did not regain fat synthesis, but that it can be switched off in some environmental settings. We then compared DNA sequence variation and protein domains of several more distantly related parasitoid species thought to have lost lipogenesis, and found no evidence for non-functionality of key lipogenesis genes. This suggests that other parasitoids may also show plasticity of fat synthesis. Last, we used individual-based simulations to show that a switch for plastic expression can remain functional in the genome for thousands of generations, even if it is only used sporadically. The evolution of plasticity could thus also explain other examples of apparent reverse evolution.

## Introduction

There are numerous cases where a complex adaptation has been lost in the evolutionary history of a lineage^1^, e.g., legs in snakes, teeth in birds, and the ability to fly in ratites. If a trait is of no use for an extended period of time, it can be selected against and/or decay by genetic drift and the accumulation of deleterious mutations^2^. The last decades have seen a surge of papers reporting reverse evolution, i.e., cases in which a trait that was once lost had reappeared^3,4^. Highly cited studies include floral adaptations^5,6^, reproductive/breeding systems^7–10^, and anatomical changes^11–13^. Trait regain over extensive periods of time is an evolutionary puzzle, because mutation accumulation in underlying genetic pathways makes the re-evolution of functional activity by reverse mutations highly unlikely^13^.

Here, we scrutinize a reported case of reverse evolution: the apparent loss and regain of an essential metabolic trait, fat synthesis^14,15^. Fat is synthesized when a surplus of sugars (and other carbohydrates) is available in the diet^16^, providing an energy reserve for future use. Fat is critical for survival and reproduction in nearly all living organisms; hence underlying metabolic and genetic pathways for fat synthesis are typically highly conserved from bacteria to humans^17–20^. A comparative study in 2010 revealed that parasitic wasps (i.e., hymenopteran parasitoids) lost the ability to synthesize fat in their common ancestor more than 200 million years ago^15,21^. The loss of fat synthesis was thought to result from consumption of host lipids, because parasitic wasps feed on a single host insect to complete their own development^22^. By consuming the fat stores of their host, fat synthesis in wasps could have become redundant and/or too costly to maintain. While the majority of parasitic wasps were found to lack lipid synthesis, fat synthesis re-appeared independently on at least three separate occasions throughout the wasp phylogeny. Further analysis revealed that fat synthesis re-evolved, particularly in species with wide host ranges, i.e., generalists^15^. Fat synthesis would be essential for these species, because generalists may not be able to manipulate their host's physiology, and fat content of the wasp depends on the fat availability within the host species utilized^15^.

In more recent years, some reports on the ability for fat synthesis in parasitic wasps contradicted previous findings. For example, several wasp species in the genus *Nasonia*^23^, thought to have lost the ability for fat synthesis^15,24^, were found capable of synthesizing fat. For yet another wasp species, the generalist *Leptopilina heterotoma*, more detailed work revealed variation in fat synthesis at the population-level—some field-collected populations synthesized fat, while others did not^25^. Moreover, there was virtually no genetic differentiation between these geographically distinct populations^25^, making a mutational basis for the loss and regain of this trait unlikely for this species.

Variation in fat synthesis between populations could be explained by adaptive plasticity, where the environmental cue used for the expression of fat synthesis is the fat content of the host^25^. We hypothesized that fat synthesis in parasitic wasps, and other parasitoids, was not lost and regained due to mutational changes in the metabolic pathway (mutation-based local adaptation), but rather that fat synthesis shows plastic expression (on or off) in response to the local environment (adaptive plasticity hypothesis). Here, we first confirm previous findings that fat synthesis can vary at the population-level^25,26^. We then use the same populations to explicitly test for plasticity of fat synthesis using a split-brood family design and stable isotope tracing, showing that fat synthesis is indeed plastic. As fat synthesis was previously thought to have been lost repeatedly in parasitoid wasps, flies, and beetles, we used the sequenced genomes of distinct parasitoid insects to evaluate whether protein domains of key lipogenesis genes have remained intact. The absence of stop codons suggests that other parasitoids could also have evolved an on–off type plasticity of fat synthesis. Last, phenotypic plasticity can remain hidden and unused for extensive periods of time. This could indeed apply to parasitoids that generally feed on lipid-rich hosts, but also to other plastic traits that are used only sporadically. Using individual-based simulations, we show that the on–off switch underlying plasticity can evolve mutational robustness and be maintained in the genome for hundreds to thousands of generations.

## Results

### A family-based experimental design reveals that fat synthesis is plastic

We first tested whether wasps could switch lipogenesis on when development occurred on a lean host. To this end, we first let females from different populations of *L. heterotoma* develop on two naturally co-occurring host species: fat-poor (“lean”) *Drosophila simulans* and fat-rich (“fat”) *D. melanogaster* (containing 63 ± 3 μg and 91 ± 4 μg, mean ± 1se storage fat, respectively; F_1,17_ = 35.95; *p* < 0.0001)*.* For each independent experiment, fat synthesis was estimated at the population-level using gravimetry (based on weight measurements), where fat content of fed females was compared to fat content of females at emergence; hence different individuals are compared. When wasps developed on lean *D. simulans*, fat synthesis had occurred in 2 out of 4 wasp populations (Table 1A). In contrast, wasps did not significantly increase their fat content when developing on fat *D. melanogaster* (Table 1B). These results confirm previous findings^25^, and suggest that wasp fat synthesis depends on the host environment.

**Table 1:** Wasps synthesize fat in a fat-poor environment.

Population	A: Development on *D. simulans*				B: Development on *D. melanogaster*			
	Sample size	Emerged	Fed	*p*-value	Sample size	Emerged	Fed	*p*-value
Belgium 1	10	17.50 ± 6.84	43.50 ± 11.38	0.043	38	36.00 ± 2.54	40.00 ± 3.22	0.336
Belgium 2	38	15.60 ± 1.02	36.83 ± 4.86	< 0.001*	32	38.50 ± 4.24	43.91 ± 3.45	0.331
UK 1	21	24.20 ± 1.49	30.91 ± 3.22	0.142 (^)	29	40.00 ± 4.41	44.30 ± 2.09	0.375
UK 2	–	–	–	–	17	33.60 ± 3.82	39.50 ± 1.50	0.522
Japan	20	12.20 ± 1.55	24.80 ± 4.86	**0.011 (^)**	13	29.17 ± 6.27	28.67 ± 8.19	0.964

Mean absolute fat amount ± 1se (in μg) was quantified in adult wasps from field-caught *L. heterotoma*. Independent experiments were performed for each population and on each host species (lean *D. simulans*, A; fat *D. melanogaster*, B) comparing fat content of recently emerged females with females that fed for 7 days. *P*-values reveal whether 7 days of feeding led to a significant overall increase (after correction for multiple testing; in bold) in fat content compared to teneral fat levels at emergence, indicating that fat synthesis had occurred. Two of the four populations tested on *D. simulans* showed increased fat content and fat synthesis on the lean host, but not on the fat host. T-tests were performed when data was normally distributed and variances equal with (^) or without log transformation. Welch’s t-test was used when variances remained unequal (*). Population UK2 was not available for testing on *D. simulans.*

**Figure 1:**
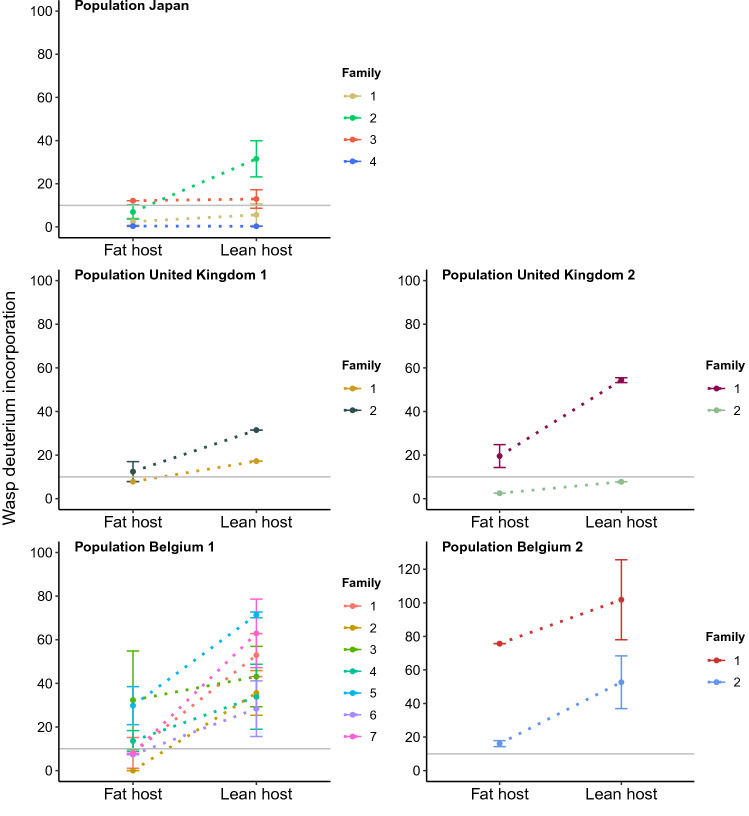
Phenotypic plasticity in five field-caught wasp populations. Incorporation of stable isotopes (%) into the fatty acid fraction (summed for C16:0, C16:1, C18:0, C18:1, and C18:2), averaged for the offspring from 17 families (± standard error; n = 138) developing in a fat-rich environment (fat host *Drosophila melanogaster*, left in each graph) and in a fat-poor environment (lean host *D. simulans*, right in each graph). The horizontal gray line indicates the threshold above which active lipogenesis takes place.

The population-level comparison of wasp fat content described above is a common, but crude measure that does not alway detects the occurrence of fat synthesis reliably. Even in the case of active fat synthesis, fat content can stay constant or even decrease if, for example, fats are burned at a faster rate than at which they are produced^27^. This means that equal or decreasing fat amounts for a group of individuals do not necessarily conclusively indicate a lack of lipogenesis. To unequivocally demonstrate that fat synthesis can be induced plastically, we turned to stable isotope tracing followed by GC–MS (Gas Chromatography-Mass Spectrometry) analyses, as in Visser et al. 2012 and 2017^28,28^. Incorporation of stable isotopes after feeding is directly dependent on fat synthesis, where stable isotopes are traced into the fatty acid fraction. An increase in stable isotope levels compared to controls (without access to stable isotopes; showing baseline incorporation of naturally occurring isotopes) thus demonstrates active lipogenesis, even if lipids are burned. To test for plasticity, we used a split-brood family design where daughters of a single mother (sharing 75% of their genome) were allowed to develop on either lean *D. simulans* or fat *D. melanogaster*. Using seventeen families belonging to four field-caught and one laboratory-based population, we showed that lipogenesis was activated in the fat-poor environment (*D. simulans*), and deactivated in the fat-rich environment (*D. melanogaster*) (Fig. 1; Table 2). These results confirm that fat synthesis is indeed a plastic trait that is generally induced in response to low host fat content and shut off in response to high host fat content. Notice also that the 17 families strongly differ in their environmental response, both in the amount of fat synthesis (on fat *D. melanogaster*) and in the slopes of their reaction norms, suggesting that there is genetic variation for plasticity. This is the first conclusive evidence that fat synthesis is plastic in *L. heterotoma*.

**Table 2:** Fat synthesis is induced plastically based on host fat content.

Fixed effects	Estimate	Std. error	t-value	*p*-value
Intercept	2.135	0.354	6.036	**0.001**
Host *D. simulans*	1.121	0.242	4.633	**0.001**

Random factors	Variance	Std. dev	*p*-value	
Population/Family (intercept)	0.274	0.523	**0.003**	

We analysed the data presented in Fig. 1 in the main text statistically by means of a linear mixed effects model (GLMM, lme4 package^69^) with host (lean *D. simulans* and fat *D. melanogaster*) and experiment (this experiment was conducted twice) as fixed effects, and family nested within population (Japan, United Kingdom 1 and 2, Belgium 1 and 2) as random effect. The percentage of incorporation of stable isotopes was used as dependent variable (n = 138), where % incorporation was summed for all fatty acids. The non-significant term experiment was sequentially removed from the model to obtain the minimal adequate model as reported in the table. Significant terms are highlighted in bold. When referring to “families,” we are referring to the comparison of daughters of singly inseminated females, which (in these haplodiploid insects) share 75% of their genome.

It has not escaped our attention that using two host species to test for the induction of lipogenesis may blur the causal mechanism underlying plasticity of fat synthesis. If host lipid content drives plasticity, we expected wasps to also start synthesizing fat when reared on lean *D. melanogaster.* To test this, we repeated the population-level experiment reported in Table 1, but now using lean *D. melanogaster*. By reducing the sugar content in the diet of *D. melanogaster*, we were able to generate leaner flies (i.e., pupae containing 52 ± 3 μg storage lipids, mean ± 1se, compared to 91 ± 4 μg storage lipids, mean ± 1se; F_1,22_ = 71.18, *p* < 0.0001). Similar to findings for *D. simulans,* populations synthesized fat on lean *D. melanogaster* hosts (except the population from Japan; Table 3). We thus conclude that plastic fat synthesis is induced by host fat content, rather than other traits differing between *D. melanogaster* and *D. simulans.*

**Table 3:** Plasticity irrespective of host species identity.

Population	Sample size	Emerged	Fed	*p*-value
Belgium 2	31	27.40 ± 1.82	38.08 ± 3.50	**0.018 (^)**
UK 1	33	25.09 ± 2.51	40.50 ± 2.66	** < 0.001**
UK 2	35	27.25 ± 2.60	38.62 ± 2.70	**0.006**
Japan	31	34.70 ± 2.72	34.36 ± 3.69	0.954

Mean absolute fat amount ± 1se (in μg) was quantified in adult wasps from field-caught and laboratory-based *L. heterotoma* populations raised on fat-poor *D. melanogaster* at two time points during adult life (Emerged: just after emergence; Fed: having fed for 7 days after emergence). *P*-values (corrected for multiple testing) reveal that fat synthesis took place in three of the four wasp populations (in bold), meaning that lipogenesis is also plastic when measured using the same host strain. T-tests were performed when data was normally distributed and variances equal with (^) or without log transformation. Population Belgium 1 was not available for testing on lean *D. melanogaster.*

### Protein domains of key lipogenic genes are functional in parasitoid wasps, a beetle and a fly

**Figure 2:**
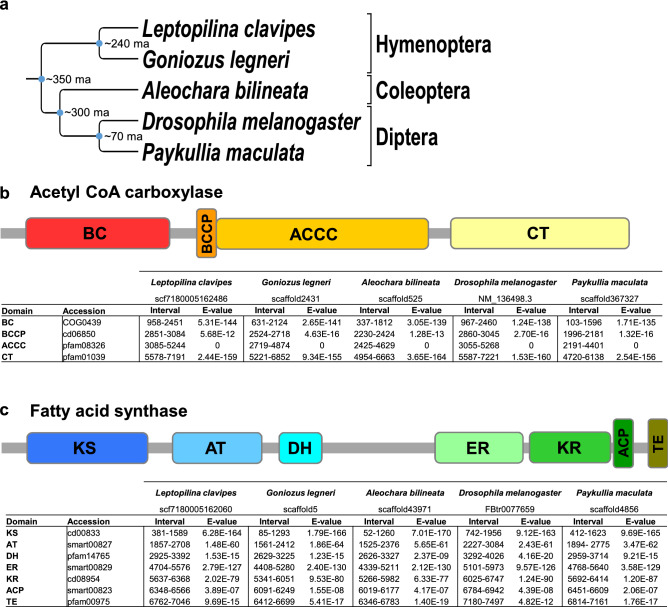
Conservation of two genes crucial for fatty acid synthesis in four parasitoid insects that supposedly had lost lipogenic activity. Long evolutionary divergence times (up to 350 MA) separate the insect *Drosophila melanogaster* (that synthesizes lipids constitutively) and 4 parasitoid insects that were assumed to have lost the ability to synthesize lipids (**a**). Acetyl coenzyme A carboxylase (ACC) and fatty acid synthase (FAS) are two essential genes for the production of fatty acids: the presence of all domains of ACC (**b**) and FAS (**c**) genes from *D. melanogaster* in the four parasitoid genomes reveals that the functional coding sequence of the two genes is conserved in these insects. A table containing the detailed length and position of the different functional domains forming the two genes, as well as conservation level of the nucleotide sequence of the domains (e-values; the lower the e-value, the higher the significance of the match) are shown for each species. Abbreviations: BC = Biotin carboxylase; BCCP = Biotin carboxyl carrier protein; ACCC = Acetyl-coA carboxylase central region; CT = Carboxyl transferase domain; KS = Ketoacyl synthase; AT = Acyl transferase; DH = Dehydratase; ER = Enoyl reductase; KR = Ketoacyl reductase; ACP = Acyl carrier protein; TE = Thioesterase. Accession numbers refer to the conserved domain identifier on NCBI’s Conserved Domain Database. Parasitoid transcript identifiers are provided underneath each species name.

The ability to synthesize fat when development takes place in a low-fat environment indicates that key genes for fat synthesis have not lost their functionality, at least in *Leptopilina heterotoma* (Visser et al. 2018^25^; this paper) and *Leptopilina boulardi*^26^. The question now is whether other parasitoids thought to have lost fat synthesis, may also show plasticity. If this is the case, we would at least expect functionality of key lipogenesis enzymes. Making use of the fact that the molecular pathway underlying fatty acid synthesis is highly conserved across animal taxa^17–20^, we conducted a comparative analysis of coding sequences of acetyl coenzyme A carboxylase (ACC)^29^ and fatty acid synthase (FAS)^19^, two enzymes that represent the only two steps of fatty acid synthesis, which in turn constitute the raw materials for stored fat (three fatty acids and a glycerol, i.e., triglycerides^30^). We used the *acc* and *fas* gene coding sequences of *D. melanogaster* as a starting point, because this fly readily synthesizes fat^31,32^. Similar gene sequences were indeed found in the genome of *L. clavipes* (family Figitidae)*,* a sister species of *L. heterotoma,* and all functional domains of ACC and FAS enzymes were recovered, suggesting fully functional coding sequences in the *L. clavipes* genome (Fig. 2). We then expanded our search for *acc* and *fas* functional coding sequences and protein domains to more distantly related parasitoids previously assumed to have lost fat synthesis independently^15^: the hymenopteran *Goniozus legneri* (family Bethylidae), the dipteran *Paykullia maculata* (family Rhinophoridae), and the coleopteran *Aleochara bilineata* (family Staphilinidae)(Fig. 2). No stop codons were found in any of the protein domains and ACC and FAS amino acid sequences of all these species aligned (Supplementary Texts 1 and 2), suggesting that these two critical genes for fat synthesis have been conserved throughout the repeated evolution of parasitism in insects. Further tests on these and other parasitoids are now needed to confirm plasticity of fat synthesis at the phenotypic level, but emerging results in other parasitoid systems, e.g., *Nasonia* species^23^ and *Meteorus pulchricornis*^33^ strengthen the notion that plasticity of fat synthesis may be the rule rather than the exception in parasitoids.

### Simulation results: a switch for plastic expression of adaptive traits is maintained in the genome when rarely used

**Figure 3:**
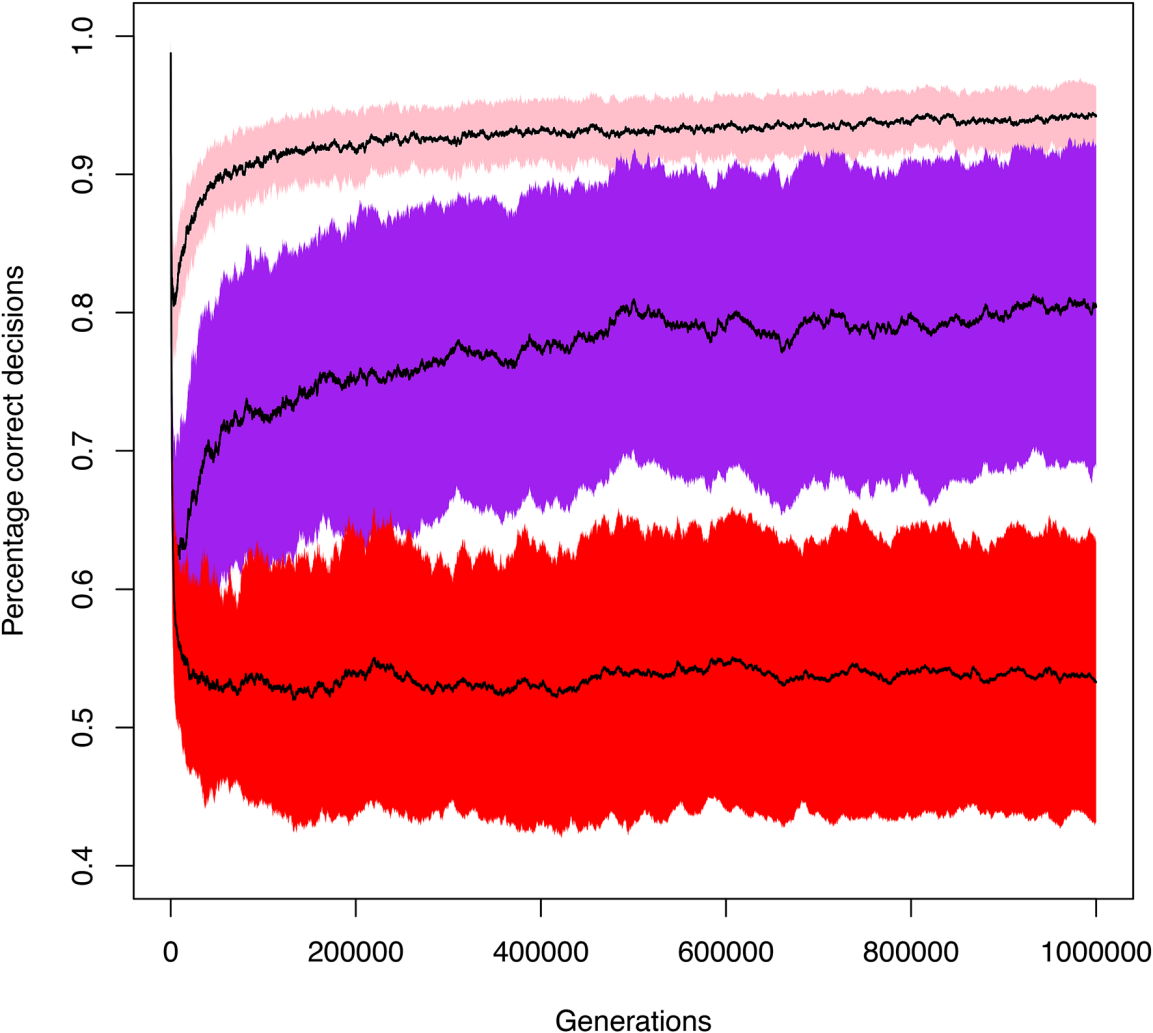
Sporadic activation is sufficient for the maintenance of adaptive plasticity. Long-term individual-based simulations showing how the performance of a gene-regulatory network (GRN) underlying adaptive plasticity changes in time when plasticity is only sporadically activated. We first evolved replicate GRNs in a variable environment where it is adaptive to switch on a metabolic pathway (fat synthesis) under low-fat conditions and to switch it off under high-fat conditions. In generation 0, a monomorphic population was established, where all N = 10,000 individuals were endowed with the same well-performing GRN (different across replicates). Subsequently, the population evolved subject to selection, mutation (μ = 0.001 per gene locus) and genetic drift in a fat-rich environment, where it is adaptive to constitutively switch off the metabolic pathway. Every 100 generations, we monitored the performance of a sample of GRNs (percentage correct decisions) in the original (fat-variable) environment: 1.0 means that the GRN is still making 100% adaptive decisions; 0.5 means that the GRN only makes 50% adaptive decision, as would be expected by a random GRN or a GRN that switches the pathway constitutively on or off. The coloured graphs show the average performance (± standard deviation) of the GRNs for three scenarios (100 replicates per simulation). Red: the population never again encounters the fat-variable environment; performance converges to 0.5, corresponding to constitutively switching off fat synthesis and hence the loss of adaptive plasticity. Pink: the individuals encounter a fat-variable environment on average every 100 generations; after an initial rapid drop in performance, a sustained high performance (> 90% correct decisions) of the GRNs is regained after about 100,000 generations. Purple: the individuals encounter a fat-variable environment on average every 1000 generations; after an initial rapid drop in performance, an intermediate performance (> 75% correct decisions) is regained gradually.

If plasticity of fat synthesis arose in the common ancestor of parasitic wasps, and wasps are generally exposed to lipid-rich hosts, the question arises whether a switching device that is not used for extensive periods of time should be lost during the course of evolution. To investigate this, we ran individual-based simulations that monitored the sustained functionality of a switching device (a gene regulatory network that could decay by mutation) that is only sporadically used in evolutionary time. Figure 3 shows that the switching device rapidly disintegrates (red simulations) if it is never used (see the methods section for model assumptions, modelling details, and simulation settings). However, even very infrequent use (pink: every 100 generations; purple: every 1000 generations) suffices to keep the switching device largely intact. Interestingly, the switching device does not erode gradually, but instead slowly evolves an improved performance over evolutionary time (i.e., the percentage of correct decisions increases with the increasing number of generations). An inspection of the evolving gene regulatory networks reveals that they become more and more robust (i.e., less and less affected by mutational decay), in line with earlier findings on network evolution^34^.

## Discussion

We provide strong support that the parasitic wasp *L. heterotoma* does not represent a case of reverse evolution at the species/population level (mutation-based adaptation), but that fat synthesis is plastic (switched on or off) in response to the environment (adaptive plasticity). In addition to experimental findings in *L. heterotoma,* we further find that key enzymes involved in fat synthesis appear to be functional, also in other parasitoid insects, including a parasitic fly and beetle originally thought to have lost the ability for fat synthesis. It can thus be expected that other parasitoid species may also show plasticity of fat synthesis. To generalize our results further, we showed that a switch underlying plastic responses evolves mutational robustness and can withstand decay if it remains unused for extensive periods of time. Another modelling study found that adaptive plasticity will be maintained in the genome for 10^8^ generations in a single populations^35^. Our simulation study shows that non-switching rapidly evolves in a fat-rich environment (leading to the loss of plasticity), but once the device has evolved mutational robustness, only incidental ‘switching on’ of the trait is sufficient for plasticity to be maintained within the genome. Plasticity itself can thus be highly robust to mutational change, which can apply also to other traits and systems. Our results further revealed large differences in the slopes of reaction norms between families, suggesting that there is genetic variation for plastic expression of fat synthesis. Plasticity of fat synthesis itself may thus evolve according to local fat availability of host populations in the wild.

The ability of *L. heterotoma* wasps to completely switch off fat synthesis, despite continued feeding on sugars, is unique and exceptional and we are unaware of a similar finding in other animals. A crucial pathway like fat synthesis is thus not constitutively expressed in parasitoids, as in other animals, but activated or deactivated in response to environmental conditions. This makes perfect sense, because these wasps typically develop on fat-rich hosts that provide all the storage fat needed by the wasps. Plasticity may still be required, however, because there is considerable spatio-temporal variation in host availability and quality. *L. heterotoma* is a generalist wasp that can parasitize more than ten different *Drosophila* species that differ substantially in size and fat availability^36^. Moreover, there is considerable geographic and seasonal variation in host species diversity and community composition^37^. Hosts are further patchily distributed with overlapping generations, suggesting considerable spatial variation at a local scale^36^. *Drosophila* are further well known to show large variation in starvation resistance, which is typically correlated with fat content^38^. It is therefore likely that plasticity of wasp fat synthesis is adaptive and evolved in response to variable environmental conditions in host fat content.

Our results at the population level (Tables 1, 3) revealed that, while fat synthesis is readily switched on in some populations, there are differences between host species as to which populations do and don’t synthesize fat. On lean *D. simulans,* the population from Japan started synthesizing fat, while population UK1 did not. The reverse is true for lean *D. melanogaster*, where Japan does not synthesize fat, but population UK1 does. There are several potential explanations for these differences. Suitability of *D. simulans* as a host depends on temperature, geographic origin, as well as host genotype^36^. Wasps may have had more difficulty in carrying over fat reserves in a less suitable strain. While dietary manipulation led to leaner *D. melanogaster*, the wasps were indeed still able to take over a considerably higher amount of fat compared to *D. simulans* (based on the fat content of wasps at emergence, which was on average ~ 17μg on *D. simulans* and ~ 28μg on *D. melanogaster*)*.* We further expect that the switch for fat synthesis depends on the genotype of the wasp. For the population-level experiments, the threshold at which fat synthesis is switched on or not likely depends on the subsample of the population that was used for testing. This is indeed confirmed by our GC-MS results that suggest that there is genetic variation for plasticity. Future experiments should aim to determine exactly how the wasp’s genotype affects the threshold at which fat synthesis is switched on or off.

Why did we not discover plasticity of fat synthesis in parasitic wasps before? First, in the 2010 study^15^, we based our experimental approach on the knowledge available in the literature at that time^14^. Up to then, all parasitoid species tested at the adult stage showed a lack of lipogenesis, including a radiotracer study in *E. vuilletti* that confirmed (similar to our stable isotope tracing method) that lipogenesis was not taking place^39^. Many subsequent and independent experiments repeatedly showed a lack of lipogenesis^14^. This led to the hypothesis that lipogenesis was lost as a consequence of the parasitoid lifestyle, which we subsequently tested by performing experiments on many different species. Second, up to now all tests for lipogenesis were done using a single host species. The 2010 study revealed that the majority of wasp species did not switch fat synthesis on, while some species, including *L. heterotoma* did. Only our later experiments, using both different host and *L. heterotoma* strains, revealed variation in lipogenic ability at the population level^25^. Third, we generally used a population-level measure (gravimetry and comparisons of groups of individuals) to infer whether a population/species was able to synthesize fat or not^15^. This method cannot distinguish between stable lipid loss (i.e., lipids are burned at a faster rate than at which they are produced^27^) and a complete lack of lipogenesis. We already recognized this earlier and developed the stable isotope technique, where we obtained similar findings between the gravimetric and stable isotope methods (but again measured on wasps reared on the same host strain^24^). This study is the first to use an integrative experimental design to test for plasticity, using hosts that vary in fat content and genetically similar wasp families.

Documented cases of trait regain over long evolutionary time, in addition to the regain of fat synthesis in parasitoids^15^, include the regaining of wings in stick insects^40^, the evolution of sexual reproduction from asexuality in mites^9^, among other examples^11^. These cases were all based on phylogenetic analyses. Such analyses were already shown to be problematic, because phylogenies do not necessarily provide a reliable representation of trait evolution^41–44^. Our results suggest that macro-evolutionary patterns of trait reversals may in fact reflect trait plasticity: the trait is not “lost” or “regained” but is rather switched off or on, depending on environmental conditions. Intriguingly, such a regulatory switch can remain largely intact, even if it is only sporadically activated (Fig. 3). We consider it plausible that our findings are not restricted to fat metabolism in parasitoid wasps: the plastic regulation of trait expression could explain more cases of apparent trait loss and reappearance at macro-evolutionary time scales. Wing formation, for example, is often observed as an atavism (the sporadic occurrence of an ancestral phenotype) in otherwise wingless insects^45^, and wing polymorphism, i.e., plasticity of wing development, is common in insects in general^46^. Similarly, many asexual populations sporadically produce sexually reproducing individuals and plasticity in reproductive mode has evolved in several organisms^47–49^. Reptiles, including turtles and lizards, represent an example in other animals, where sex can be determined by the environment (temperature) or be under genetic control^50^. Altogether these examples suggest that plasticity could be a common principle explaining apparent reverse evolution.

Phenotypic plasticity is ubiquitous among most living organisms, but despite its frequent appearance in nature we still know relatively little about the processes and mechanisms underlying plastic trait expression^51,52^. A recent study by Ho et al^53^ revealed that plastic transcriptomic responses can play a critical role when organisms are re-exposed to ancestral environments. They show that plasticity observed in chickens, bacteria, and guppies, allows fast re-adaptation to previously encountered environments. When exposed to new, generally stressful environments, plasticity has been suggested to be at the base of generating novel, complex phenotypes. This plasticity-led evolution hypothesis states that organisms first respond to environmental change plastically, which can be selected upon if there is genetic variation for plasticity^53–56^. Depending on the new environment, plasticity can be favoured and selected upon leading to increased environmental sensitivity or selected against leading to canalization and genetic assimilation^57,58^. Based on the findings described in this paper, *L. heterotoma* represents an exceptional model system to address key gaps of knowledge in plasticity research based on natural populations. The use of natural populations is particularly important to study in the context of rapid global environmental change^59^.

## Methods

### Experimental study and protein domain analysis

#### Insects

Hosts and parasitoids were maintained as previously described^25^. Five *Leptopilina heterotoma* (Hymenoptera: Figitidae) populations were used for experiments: a population from Japan (Sapporo), two populations from the United Kingdom (1: Whittlesford; 2: Great Shelford) and two populations from Belgium (1: Wilsele; 2: Eupen). Information on collection sites, including GPS coordinates, can be found in^25^.

#### Determination of host fat content

*D. simulans* and *D. melanogaster* hosts were allowed to lay eggs during 24 h in glass flasks containing ~ 50 mL standard medium^25^. After two days, developing larvae were sieved and ~ 200 were larvae placed in a *Drosophila* tube containing ~ 10 mL medium. Seven days after egg laying, newly formed pupae were frozen at – 18 °C, after which fat content was determined as described in^25^, where dry weight before and after neutral fat extraction was used to calculate absolute fat amount (in μg) for each host. The host pupal stage was chosen for estimating fat content, because at this point the host ceases to feed, while the parasitoid starts consuming the entire host^36^. All data were analysed using R Project version 3.4.3^60^. Fat content of hosts was compared using a one-way ANOVA with host species as fixed factor.

#### Manipulation of host fat content

To generate leaner *D. melanogaster* hosts, we adapted our standard food medium^25^ to contain 100 times less (0.5 g) sugar per litre water. Manipulating sugar content did not alter the structure of the food medium, thus maintaining similar rearing conditions, with the exception of sugar content. Fat content of leaner and fatter *D. melanogaster* hosts was determined and analysed as described above.

#### Fat synthesis quantification of wasp populations

Mated female *L. heterotoma* were allowed to lay eggs on host fly larvae collected as described above with ad libitum access to honey as a food source until death. Honey consists of sugars and other carbohydrates that readily induce fat synthesis. After three weeks, adult offspring emergence was monitored daily and females were haphazardly placed in experimental treatments. Females were either killed at emergence (to measure teneral lipid reserves) or after feeding for 7 days on honey. Wasps were frozen at − 18 °C after completion of experiments. Fat content was determined as described above for hosts. The ability for fat synthesis was then determined by comparing mean fat levels of recently emerged compared to fed individuals, similar to procedures described in^15,25,28^. An increase in fat levels after feeding is indicative of active fat synthesis; equal or lower fat levels suggest fat synthesis did not take place. Each population tested on *D. melanogaster* or *D. simulans* represented an independent dataset that was analysed separately, as in Visser et al. 2018^25^, because we are interested in the response of each population on each host species. We used T-tests when data was normally distributed and variances equal, log-transformed data for non-normal data, and a Welch's t-test when variances were unequal. We corrected for multiple testing using Benjamini and Hochberg’s False Discovery Rate^61^.

#### Fat synthesis quantification using a familial design and GC–MS analyses

To tease apart the effect of wasp genotype and host environment, we used a split-brood design where the offspring of each mother developed on lean *D. simulans* or fat *D. melanogaster* hosts in two replicated experiments (experiment 1 and 2). In both experiments, mothers were allowed to lay eggs in ~ 200 2nd to 3rd instar host larvae of one species for four days, after which ~ 200 host larvae of the other species were offered during four days. The order in which host larvae were presented was randomized across families. Following offspring emergence, daughters were allocated into two treatment groups: a control where females were fed a mixture of honey and water (1:2 w/w) or a treatment group fed a mixture of honey and deuterated water (Sigma Aldrich) (1:2 w/w; stable isotope treatment) for 7 days. Samples were prepared for GC–MS as described in ^28^. Incorporation of up to three deuterium atoms can be detected, but percent incorporation is highest when only 1 deuterium atom is incorporated. As incorporation of a single atom unequivocally demonstrates active fat synthesis, we only analysed percent incorporation (in relation to the parent ion) for the abundance of the m + 1 ion. Percent incorporation was determined for five fatty acids, C16:1 (palmitoleic acid), C16:0 (palmitate), C18:2 (linoleic acid), C18:1 (oleic acid), and C18:0 (stearic acid), and the internal standard C17:0 (margaric acid). Average percent incorporation for C17:0 was 19.4 (i.e. baseline incorporation of naturally occurring deuterium) and all values of the internal standard remained within 3 standard deviations of the mean (i.e. 1.6). Percent incorporation of control samples was subtracted from treatment sample values to correct for background levels of deuterium (i.e. only when more deuterium is incorporated in treatment compared to controls fatty acids are actively being synthesized). For statistical analyses, percent incorporation was first summed for C16:1, C16:0, C18:2, C18:1 and C18:0 to obtain overall incorporation levels, as saturated C16 and C18 fatty acids are direct products of the fatty acid synthesis pathway (that can subsequently be desaturated).

Data (presented in Fig. 1) was analysed by means of a linear mixed effects model (GLMM, lme4 package) with host (lean *D. simulans* and fat *D. melanogaster*) and experiment (conducted twice) as fixed effect, family nested within population (Japan, United Kingdom 1 and 2, Belgium 1 and 2) as random factor, and percentage of incorporation of stable isotopes as dependent variable (log transformed; n = 138). Non-significant terms (i.e., experiment) were sequentially removed from the model to obtain the minimal adequate model as reported in Table 2. When referring to “families,” we are referring to the comparison of daughters of singly inseminated females, which (in these haplodiploid insects) share 75% of their genome.

### Identification of functional *acc* and *fas* genes in distinct parasitoid species

To obtain *acc* and *fas* nucleotide sequences for *L. clavipes, G. legneri, P. maculata* and *A. bilineata*, we used *D. melanogaster* mRNA ACC transcript variant A (NM_136498.3 in Genbank) and FASN1-RA (FBtr0077659 in FlyBase) and blasted both sequences against transcripts of each parasitoid (using the blast function available at http://www.parasitoids.labs.vu.nl^62,63^). Each nucleotide sequence was then entered in the NCBI Conserved Domain database^64^ to determine the presence of all functional protein domains. All sequences were then translated using the Expasy translate tool (https://web.expasy.org/translate/), where the largest open reading frame was selected for further use and confirming no stop codons were present. Protein sequences were then aligned using MAFFT v. 7 to compare functional amino acid sequences between all species (Supplementary files 1 and 2)^65^.

### Simulation study

We consider the general situation where phenotypic plasticity is only sporadically adaptive and ask the question whether and under what circumstances plasticity can remain functional over long evolutionary time periods when the regulatory processes underlying plasticity are gradually broken down by mutations. We consider a regulatory mechanism that switches on or off a pathway (like fat synthesis) in response to environmental conditions (e.g., host fat content).

#### Fitness considerations

We assume that the local environment of an individual is characterized by two factors: fat content *F* and nutrient content *N*, where nutrients represent sugars and other carbohydrates that can be used to synthesize fat. Nutrients are measured in units corresponding to the amount of fat that can be synthesized from them. We assume that fitness (viability and/or fecundity) is directly proportional to the amount of fat stored by the individual. When fat synthesis is switched off, this amount is equal to *F*, the amount of fat in the environment. When fat synthesis is switched on, the amount of fat stored is assumed to be $N - c + (1 - k)F$. This expression reflects the following assumptions: *(i)* fat is synthesized from the available nutrients, but this comes at a fitness cost *c*; *(ii)* fat can still be absorbed from the environment, but at a reduced rate $(1 - k)$. It is adaptive to switch on fat synthesis if $N - c + (1 - k)F$ is larger than *F*, or equivalently if $F < \tfrac{1}{k}(N - c)$.

**Figure 4:**
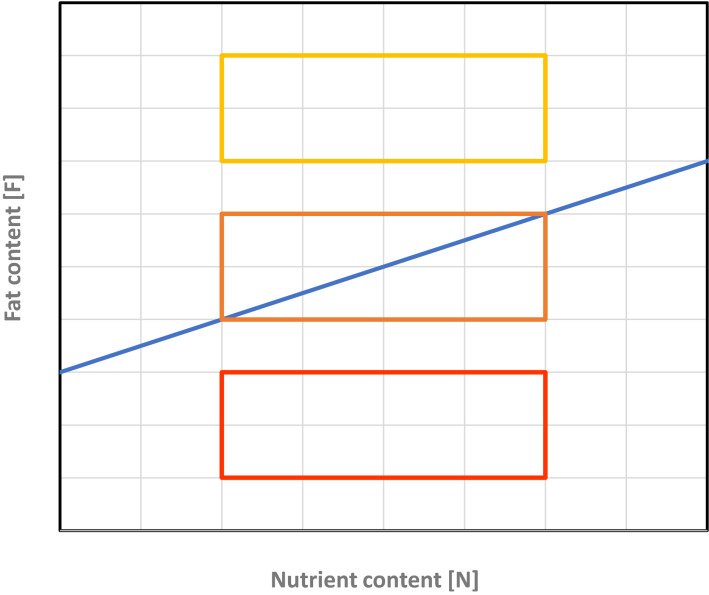
Environmental conditions encountered by the model organisms. For a given combination of environmental nutrient content *N* and environmental fat content *F*, it is adaptive to switch on fat synthesis if (*N*, *F*) is below the blue line (corresponding to $F < \tfrac{1}{k}(N - c)$) and to switch it off otherwise. The three boxes illustrate three types of environment: a low-fat environment (red) where fat synthesis should be switched on constitutively; a high-fat environment (yellow) where fat synthesis should be switched off constitutively; and an intermediate-fat environment (orange) where a plastic switch is selectively favoured.

The right-hand side of this inequality is a straight line, which is illustrated by the blue line in Fig. 4. The three boxes in Fig. 4 illustrate three types of environmental conditions.

- *Red box* low-fat environments. Here, $F < \tfrac{1}{k}(N - c)$ is always satisfied, implying that fat synthesis should be switched on constitutively.
- *Yellow box* high-fat environments. Here, $F > \tfrac{1}{k}(N - c)$, implying that fat synthesis should be switched off constitutively.
- *Orange box* intermediate-fat environments. Here, fat synthesis should be plastic and switched on if for the given environment (*N*, *F*) the fat content is below the blue line and switched off otherwise.

The simulations reported here were all run for the parameters $k = \tfrac{1}{2}{\text{ and }}c = \tfrac{1}{4}$. We also investigated many other combinations of these parameters; in all cases, the results were very similar to those reported in Fig. 3.

#### Gene regulatory networks (GRN)

In our model, the switching device was implemented by an evolving gene regulatory network (as in van Gestel and Weissing^66^). The simulations shown in Fig. 3 of the main text are based on the simplest possible network that consists of two receptor nodes (sensing the fat and the nutrient content in the local environment, respectively) and an effector node that switches on fat synthesis if the combined weighted input of the two receptor nodes exceeds a threshold value *T* and switches it off otherwise. Hence, fat synthesis is switched on if $w_{F} F + w_{N} N > T$ (and off otherwise). The GRN is characterized by the weighing factors $w_{F} {\text{ and }}w_{N}$ and the threshold *T*. These parameters are transmitted from parents to offspring, and they evolve subject to mutation and selection. We also considered alternative network structures (all with two receptor nodes and one effector node, but with a larger number of evolvable weighing factors^67^, and obtained very similar results, see below).

For the simple GRN described above, the switching device is 100% adaptive when the switch is on (i.e., $w_{F} F + w_{N} N > T$) if $F < \tfrac{1}{k}(N - c)$ and off otherwise. A simple calculation yields that this is the case if: $w_{N} > 0{, }w_{F} = - k{\kern 1pt} w_{N} {\text{ and }}T = c{\kern 1pt} w_{N}$.

#### Evolution of the GRN

For simplicity, we consider an asexual haploid population with discrete, non-overlapping generations and fixed population size $N = 10,000$. Each individual has several gene loci, each locus encoding one parameter of the GRN. In case of the simple network described above, there are three gene loci, each with infinitely many alleles. Each individual harbours three alleles, which correspond to the GRN parameters $w_{F} {, }w_{N} {\text{ and }}T$, and hence determine the functioning of the genetic switch. In the simulations, each individual encounters a randomly chosen environment $(N{, }F)$. Based on its (genetically encoded) GRN, the individual decides on whether to switch on or off fat synthesis. If synthesis is switched on, the individual’s fitness is given by $N - c + (1 - k)F$; otherwise its fitness is given by *F*. Subsequently, the individuals produce offspring, where the number of offspring produced is proportional to the amount of fat stored by an individual. Each offspring inherits the genetic parameters of its parent, subject to mutation. With probability μ (per locus) a mutation occurs. In such a case the parental value (in case of a simple network: the parent’s allelic value $w_{F} {, }w_{N} {\text{ or }}T$) is changed to a mutated value ($w_{F} { + }\delta {, }w_{N} { + }\delta {\text{ or }}T + \delta$), where the mutational step size δ is drawn from a normal distribution with mean zero and standard deviation σ. In the reported simulations, we chose $\mu = 0.001$ and $\sigma = 0.1$. The speed of evolution is proportional to $\mu \cdot \sigma^{2}$, implying that the rate of change in Fig. 3 (both the decay of plasticity and the rate of regaining adaptive plasticity) are positively related to μ and σ.

#### Preadaptation of the GRNs

Starting with a population with randomly initialized alleles for the GRN parameters, we first let the population evolve for 10,000 generations in the intermediate-fat environment (the orange box in Fig. 4). In all replicate simulations, a “perfectly adapted switch” (corresponding to $w_{N} > 0{, }w_{F} = - k{\kern 1pt} w_{N} {\text{ and }}T = c{\kern 1pt} w_{N}$) evolved, typically within 1,000 generations. Still, the evolved GRNs differed across replicates, as they evolved different values of $w_{N} > 0$. These evolved networks were used to seed the populations in the subsequent “decay” simulations.

#### Evolutionary decay of the GRNs

For the decay experiments reported in Fig. 3 of the main text, we initiated a large number of monomorphic replicate populations with one of the perfectly adapted GRNs from the preadaptation phase. These populations were exposed for an extended period of time (1,000,000 generations) to a high-fat environment (the yellow box in Fig. 4), where all preadapted GRNs switched off fat synthesis. However, in some scenarios, the environmental conditions changed back sporadically (with probability *q*) to the intermediate-fat environment (the orange box in Fig. 4), where it is adaptive to switch on fat metabolism in 50% of the environmental conditions (when (*N*, *F*) is below the blue line in Fig. 4). In Fig. 3, we report on the changing rates $q = 0.0$ (no changing back; red), $q = 0.001$ (changing back once every 1,000 generations; purple), and $q = 0.01$ (changing back once every 100 generations; pink). When such a change occurred, the population was exposed to the intermediate-fat environment for *t* generations (Fig. 3 is based on *t* = 3).

Throughout the simulation, the performance of the network was monitored every 100 generations as follows: 100 GRNs were chosen at random from the population, and each of these GRNs was exposed to 100 randomly chosen environmental conditions from the intermediate-fat environment (orange box in Fig. 4). From this, we could determine the average percentage of “correct” decisions (where the network should be switched on if and only if $F < \tfrac{1}{k}(N - c)$. 1.0 means that the GRN is still making 100% adaptive decisions; 0.5 means that the GRN only makes 50% adaptive decision, as would be expected by a random GRN or a GRN that switches the pathway constitutively on or off. This measure for performance in the “old” intermediate-fat environment was determined for 100 replicate simulations per scenario and plotted in Fig. 3 (mean ± standard deviation).

#### Evolving robustness of the GRNs

The simulations in Fig. 3 are representative for all networks and parameters considered. Whenever $q = 0.0$, the performance of the regulatory switch eroded in evolutionary time, but typically at a much lower rate in case of the more complex GRNs. Whenever $q = 0.01$, the performance of the switch went back to levels above 90% and even above 95% for the more complex GRNs. Even for $q = 0.001$, a sustained performance level above 75% was obtained in all cases.

Intriguingly, in the last two scenarios the performance level first drops rapidly (from 1.0 to a much lower level, although this drop is less pronounced in the more complex GRNs) and subsequently recovers to reach high levels again. Apparently, the GRNs have evolved a higher level of robustness, a property that seems to be typical for evolving networks^8^. For the simple GRN studied in Fig. 3, this outcome can be explained as follows. The initial network was characterized by the genetic parameters $w_{N} > 0{, }w_{F} = - k{\kern 1pt} w_{N} {\text{ and }}T = c{\kern 1pt} w_{N}$ (see above), where $w_{N}$ was typically a small positive number. In the course of evolutionary time, the relation between the three evolving parameters remained approximately the same, but $w_{N}$ (and with it the other parameters) evolved to much larger values. This automatically resulted in an increasingly robust network, since mutations with a given step size distribution affect the performance of a network much less when the corresponding parameter is large in absolute value.

#### Costs of plasticity

Phenotypically plastic organisms can incur different types of costs^68^. In our simple model, we only consider the cost of phenotype-environment mismatching, that is, the costs of expressing the ‘wrong’ phenotype in a given environment. When placed in a high-fat environment, the preadapted GRNs in our simulations take the ‘right’ decision to switch off fat metabolism. Accordingly, they do not face any costs of mismatching. Yet, the genetic switch rapidly decays (as indicated in Fig. 3 by the rapid drop in performance when tested in an intermediate-fat environment), due to the accumulation of mutations.

It is not unlikely that there are additional fitness costs of plasticity, such as the costs for the production and maintenance of the machinery underlying plasticity^68^. In the presence of such constitutive costs, plasticity will be selected against when organisms are living in an environment where only one phenotype is optimal (as in the high- and low-fat environments in Fig. 4). This would obviously affect the evolutionary dynamics in Fig. 3, but the size of the effect is difficult to judge, as the constitutive costs of plasticity are notoriously difficult to quantify. In case of the simple switching device considered in our model, we consider the constitutive costs of plasticity as marginal, but these costs might be substantial in other scenarios.

## Supplementary Information

Supplementary Information

## Data Availability

All data is available on: https://visserlab.be/download/visser-et-al-2021-experimental-data.zip and https://visserlab.be/download/visser-et-al-2021-simulation-data.zip.

## References

[1] Ellers J, Kiers T, Currie CR, Mcdonald BR, Visser B (2012). Ecological interactions drive evolutionary loss of traits. Ecol. Lett..

[2] Lahti DC (2009). Relaxed selection in the wild. Trends Ecol. Evol..

[3] Collin R, Miglietta MP (2008). Reversing opinions on Dollo’s law. Trends Ecol. Evol..

[4] Esfeld K (2019). Pseudogenization and resurrection of a speciation gene. Curr. Biol..

[5] Zufall RA, Rausher MD (2004). Genetic changes associated with floral adaptation restrict future evolutionary potential. Nature.

[6] Tripp EA, Manos PS (2008). Is floral specialization an evolutionary dead-end? Pollination system transitions in Ruellia (Acanthaceae). Evolution.

[7] Lee MSY, Shine R (1998). Reptilian viviparity and Dollo’s law. Evolution.

[8] Igic B, Bohs L, Kohn JR (2006). Ancient polymorphism reveals unidirectional breeding system shifts. Proc. Natl. Acad. Sci. U. S. A..

[9] Domes K, Norton RA, Maraun M, Scheu S (2007). Reevolution of sexuality breaks Dollo’s Law. Proc. Natl. Acad. Sci..

[10] Lynch VJ, Wagner GP (2010). Did egg-laying boas break dollo’s law? Phylogenetic evidence for reversal to oviparity in sand boas (Eryx: Boidae). Evolution.

[11] Collin R, Cipriani R (2003). Dollo's Law and the Re-Evolution of Shell Coiling. Proc. Biol. Sci..

[12] Kohlsdorf TIK, Wagner GP (2006). Evidence for the reversibility of digit loss: a phylogenetic study of limb evolution in *Bachia* (Gymnophthalmidae: Squamata). Evolution.

[13] Wiens JJ (2011). Re-evolution of lost mandibular teeth in frogs after more than 200 million years, and re-evaluating Dollo’s law. Evolution.

[14] Visser B, Ellers J (2008). Lack of lipogenesis in parasitoids: a review of physiological mechanisms and evolutionary implications. J. Insect Physiol..

[15] Visser B (2010). Loss of lipid synthesis as an evolutionary consequence of a parasitic lifestyle. Proc. Natl. Acad. Sci..

[16] Turkish AR, Sturley SL (2009). The genetics of neutral lipid biosynthesis: an evolutionary perspective. Am. J. Physiol. Endocrinol. Metab..

[17] Jenke-kodama H, Sandmann A, Müller R, Dittmann E (2005). Evolutionary implications of bacterial polyketide synthases. Mol. Biol. Evol..

[18] Maier T, Leibundgut M, Ban N (2008). The crystal structure of a mammalian fatty acid synthase. Science.

[19] Maier T, Leibundgut M, Boehringer D, Ban N (2010). Structure and function of eukaryotic fatty acid synthases. Q. Rev. Biophys..

[20] Bukhari HST, Jakob RP, Maier T (2014). Evolutionary origins of the multienzyme architecture of giant fungal fatty acid synthase. Structure.

[21] Peters RS (2017). Evolutionary history of the Hymenoptera. Curr. Biol..

[22] Godfray HCJ (1994). Parasitoids: Behavioural and evolutionary ecology.

[23] Prager L, Bruckmann A, Ruther J (2019). De *novo* biosynthesis of fatty acids from α-D-glucose in parasitoid wasps of the *Nasonia* group. Insect Biochem. Mol. Biol..

[24] Visser B (2012). Transcriptional changes associated with lack of lipid synthesis in parasitoids. Genome Biol. Evol..

[25] Visser B (2018). Variation in lipid synthesis, but genetic homogeneity, among *Leptopilina *parasitic wasp populations. Ecol. Evol..

[26] Moiroux J (2010). Local adaptations of life-history traits of a *Drosophila* parasitoid, *Leptopilina boulardi: * does climate drive evolution?. Ecol. Entomol..

[27] Ament SA (2011). Mechanisms of stable lipid loss in a social insect. J. Exp. Biol..

[28] Visser B, Willett DS, Harvey JA, Alborn HT (2017). Concurrence in the ability for lipid synthesis between life stages in insects. R. Soc. Open Sci..

[29] Abu-Elheiga L (2005). Mutant mice lacking acetyl-CoA carboxylase 1 are embryonically lethal. Proc. Natl. Acad. Sci. U. S. A..

[30] Wakil SJ (1989). Fatty acid synthase, a proficient multifunctional enzyme. Biochemistry.

[31] Geer BW, Langevin ML, McKechnie SW (1985). Dietary ethanol and lipid synthesis in *Drosophila melanogaster*. Biochem. Genet..

[32] Zinke I, Schütz CS, Katzenberger JD, Bauer M, Pankratz MJ (2002). Nutrient control of gene expression in *Drosophila*: microarray analysis of starvation and sugar-dependent response. EMBO J..

[33] Wang J (2020). Lipid dynamics, identification, and expression patterns of fatty acid synthase genes in an endoparasitoid, *Meteorus pulchricornis* (Hymenoptera: Braconidae). Int. J. Mol. Sci..

[34] Wagner A (2007). Robustness and Evolvability in Living Systems.

[35] Masel, J., King, O. D. & Maughan, H. The loss of adaptive plasticity during long periods of environmental stasis.* Am. Nat*. **169**, 38–46 (2007). 10.1086/510212 PMC1766558 17206583

[36] Fleury F, Gibert P, Ris N, Allemand R (2009). Ecology and life history evolution of frugivorous *Drosophila* parasitoids. Adv. Parasitol..

[37] Lue C, Borowy D, Buffington ML, Leips J (2018). Geographic and seasonal variation in species diversity and community composition of frugivorous *Drosophila* (Diptera: Drosophilidae) and their *Leptopilina* (Hymenoptera: Figitidae) parasitoids. Environ. Entomol..

[38] Hoffmann ARYA, Harshman LG (1999). Desiccation and starvation resistance in *Drosophila*: patterns of variation at the species, population and intrapopulation levels. Heredity (Edinb)..

[39] Giron D, Casas J (2003). Lipogenesis in an adult parasitic wasp. J. Insect Physiol..

[40] Whiting MF, Bradler S, Maxwell T (2003). Loss and recovery of wings in stick insects. Nature.

[41] Stone G, French V (2003). Evolution: Have wings come, gone and come again?. Curr. Biol..

[42] Goldberg EE, Igic B (2008). On phylogenetic tests of irreversible evolution. Evolution.

[43] Christin P-A, Freckleton RP, Osborne CP (2010). Can phylogenetics identify C4 origins and reversals?. Trends Ecol. Evol..

[44] Galis F, Arntzen JW, Lande R (2010). Dollo’s law and the irreversibility of digit loss in *Bachia*. Evolution.

[45] Hall BK (1984). Developmental mechanisms underlying the formation of atavisms. Biol. Rev..

[46] Zhang C-X, Brisson JA, Xu H-J (2019). Molecular mechanisms of wing polymorphism in insects. Annu. Rev. Entomol..

[47] Parker, D. J. *et al.* Repeated Evolution of Asexuality Involves Convergent Gene Expression Changes, *Mol. Biol. Evol.* **36**(2), 350–364. 10.1093/molbev/msy217. (2019). 10.1093/molbev/msy217 PMC6404633 30445505

[48] Tvedte ES, Logsdon JM, Forbes AA (2019). Sex loss in insects: causes of asexuality and consequences for genomes. Curr. Opin. Insect Sci..

[49] Hanschen ER, Herron MD, Wiens JJ, Nozaki H, Michod RE (2018). Repeated evolution and reversibility of self-fertilization in the volvocine green algae. Evolution.

[50] Janzen, F.J. & Phillips, P.C. Exploring the evolution of environmental sex determination, especially in reptiles. *J. Evol Biol* **19**, 1775–1784 (2006). 10.1111/j.1420-9101.2006.01138.x 17040374

[51] West-Eberhard M (2003). Developmental Plasticity and Evolution.

[52] Sommer RJ (2020). Phenotypic plasticity: from theory and genetics to current and future challenges. Genetics.

[53] Ho WC, Li D, Zhu Q, Zhang J (2020). Phenotypic plasticity as a long-term memory easing readaptations to ancestral environments. Sci. Adv..

[54] Levis NA, Pfennig DW (2016). Evaluating ‘plasticity-first’ evolution in nature: key criteria and empirical approaches. Trends Ecol. Evol..

[55] Levis NA, Pfennig DW (2019). Plasticity-led evolution: evaluating the key prediction of frequency-dependent adaptation. Proc. R. Soc. B Biol. Sci..

[56] Levis NA, Pfennig DW (2020). Plasticity-led evolution: a survey of developmental mechanisms and empirical tests. Evol. Dev..

[57] Waddington CH (1953). Genetic assimilation of an acquired character. Evolution.

[58] Suzuki Y, Nijhout HF (2006). Evolution of a polyphenism by genetic accommodation. Science.

[59] Lann L, Baaren JV, Visser B (2021). Dealing with predictable and unpredictable temperatures in a climate change context: the case of parasitoids and their hosts. J. Exp. Biol..

[60] R Development Core Team. R: A Language and Environment for Statistical Computing. (2016).

[61] Benjamini Y, Hochberg Y (1995). Controlling the false discovery rate: a practical and powerful approach to multiple testing. J. R. Statical Soc..

[62] Kraaijeveld K, Neleman P, Marien J, de Meijer E, Ellers J (2019). Genomic resources for *Goniozus legneri*, *Aleochara bilineata* and *Paykullia maculata*, representing three independent origins of the parasitoid lifestyle in insects. G3 Genes Genomes Genet..

[63] Kraaijeveld K (2016). Decay of sexual trait genes in an asexual parasitoid wasp. Genome Biol. Evol..

[64] Marchler-Bauer A (2017). CDD/SPARCLE: Functional classification of proteins via subfamily domain architectures. Nucl. Acids Res..

[65] Katoh K, Standley DM (2013). MAFFT multiple sequence alignment software version 7: improvements in performance and usability. Mol. Biol. Evol..

[66] Van Gestel J, Weissing FJ (2018). Is plasticity caused by single genes?. Nature.

[67] Van Gestel J, Weissing FJ (2016). Regulatory mechanisms link phenotypic plasticity to evolvability. Sci. Rep..

[68] Auld JR, Agrawal AA, Relyea RA (2010). Re-evaluating the costs and limits of adaptive phenotypic plasticity. Proc. R. Soc. B Biol. Sci..

[69] Bates, D., Mächler, M., Bolker, B., & Walker, S. Fitting Linear Mixed-Effects Models Using lme4.* J. Statical Software*, **67**(1), 1–48.10.18637/jss.v067.i01. (2015).

