# Supplementary material for: Phenotypic plasticity explains apparent reverse evolution of fat synthesis in parasitic wasps
Source: Sci Rep. 2021 Apr 8;11:7751. doi: 10.1038/s41598-021-86736-8 (PMC8032832; doi:10.1038/s41598-021-86736-8)
Supplement: Supplementary file 1 — Supplementary Information [file 41598_2021_86736_MOESM1_ESM.pdf]

Phenotypic plasticity explains apparent reverse evolution of fat synthesis in parasitic wasps

Bertanne Visser[1*], Hans T. Alborn[2], Suzon Rondeaux[1], Manon Haillot[1], Thierry Hance[3], Darren Rebar[4], Jana M. Riederer[5], Stefano Tiso[5], Timo J.B. van Eldijk[5], Franz J. Weissing[5] & Caroline M. Nieberding[6]

Supplementary file 1: Acetyl coenzyme A carboxylase (ACC) amino acid sequence alignment for *D. melanogaster*, *P. maculata*, *L. clavipes*, *G. legneri* and *A. bilineata* :

```
                                         10        20        30        40
50        60        70        80        90        100

....|....|....|....|....|....|....|....|....|....|....|....|....|....|....|..
..|....|....|....|....|
Dmel ACC protein transcript va  ---------------------------------------------
----------------MLITILGTL---AAFLAFLLTLIFGRGQKRSKPVQSS--
Pmac ACC protein Pmac maker-sc  ---------------------------------------------
-------------------------------------------------------
Gleg ACC protein augustus_mask
MTDQEDKSEPNLREERAAPQRKIRHRGIVEREILFPNVTESLEVAVVEDTLSILRITFEAALT.A..LA.LAC...G
VV.SRVWNASASS.GNTGYTEND
Lclav ACC protein scf718000516  ---------------------------------------------
-------------------------------------------------------
Abil ACC protein Abil maker-sc  ---------------------------------------------
----------------------------------MAKQLNR.NS..---------

                                        110       120       130       140
150       160       170       180       190       200

....|....|....|....|....|....|....|....|....|....|....|....|....|....|....|..
..|....|....|....|....|
Dmel ACC protein transcript va  ------------------AATSATTATTGDSDNGNTNHNSSV-
IAATATATTTSSKPPIAPAAPPSAVKASDKRFPKACIKKVQFSSESLRSDVDELCDQ
Pmac ACC protein Pmac maker-sc  ---------------------------------------------
-------------------------------------------------------
Gleg ACC protein augustus_mask  NSRIDRDSEQEEALNAERM.EAPVSFVV..P.---
ADP.EELE.EDSFPNE.SD.NIQMQQTIA.GLLE---R.------RRLR----------------
Lclav ACC protein scf718000516  ------------------MTET.VSFVL..P-----
DPKEELE.EDSFPE--PEANDR.QQPIL.GL.E---R.------.RLR.---------------
Abil ACC protein Abil maker-sc  -------------------------FVI..E.V.S.DN-------
--.P.DES.------.FTI..I.E-------------------------------

                                        210       220       230       240
250       260       270       280       290       300

....|....|....|....|....|....|....|....|....|....|....|....|....|....|....|..
..|....|....|....|....|
Dmel ACC protein transcript va  LKDSALSNLSNDNIRIACHQNNNN---
SSINKNQNNNSIDISISISKMSETNESNDTAAQSAEGERPSFLVGDEIDERAAEAGEACDEFPLKMQNDVRQN
Pmac ACC protein Pmac maker-sc  ---------------------------------------------
-------------------------------------------------------
Gleg ACC protein augustus_mask  ---------------------------------------------
-------------------------------------------------------
Lclav ACC protein scf718000516  ----V.AER.EK.---.AWKSSD.LLAGIVRM.SE..EDN---
EVF.REPSI.TEK..G-----------------------------------------
Abil ACC protein Abil maker-sc  ------.H.QD.D-----------------.L.G.G.GY-----
..-------------------------------------------.VW..QARLLK

                                        310       320       330       340
```

```
350       360       370       380       390       400

....|....|....|....|....|....|....|....|....|....|....|....|....|....|....|..
..|....|....|....|....|
Dmel ACC protein transcript va  GDISERRKRLRPSMSRGTGL--
GQDRHQDRDFHIATTEEFVKRFGGTRVINKVLIANNGIAAVKCMRSIRRWAYEMFKNERAIRFVVMVTPEDLKANAE
Y
Pmac ACC protein Pmac maker-sc  -------------.......--
....Y......V........K...N....R....................S....Q....V................
.
Gleg ACC protein augustus_mask  -----------
....Q..VMIQA.S.L.EK..TV..P....H..............................S.........V.....
............
Lclav ACC protein scf718000516  ---------
.T....Q..VMIQA.S.QLEK..TV..P....R.....K........................S.........V...
..............
Abil ACC protein Abil maker-sc  PGV.-----.T....Q..VI--
M.N.LHE...TV..P....R.....K....................V...S........SV................
.

                                        410       420       430       440
450       460       470       480       490       500

....|....|....|....|....|....|....|....|....|....|....|....|....|....|....|..
..|....|....|....|....|
Dmel ACC protein transcript va
IKMADHYVPVPGGSNNNNYANVELIVDIALRTQVQAVWAGWGHASENPKLPELLHKEGLVFLGPPERAMWALGDKVA
SSIVAQTAEIPTLPWSGSDLKAQ
Pmac ACC protein Pmac maker-sc
.........................................................N...................
........D.........E...H
Gleg ACC protein augustus_mask
.....Q.......T...............I..........................NNIC.I..S..........I.
........DV........E...H
Lclav ACC protein scf718000516
.....Q...................I...I..........................NNIC.I..S..........I.
........DV........E....
Abil ACC protein Abil maker-sc
.............T...........I...V.C........................NNIA.I...DK........I.
........D..............

                                        510       520       530       540
550       560       570       580       590       600

....|....|....|....|....|....|....|....|....|....|....|....|....|....|....|..
..|....|....|....|....|
Dmel ACC protein transcript va
YSGKKIKISSELFARGCVTNVEQGLAAVNKIGFPVMIKASEGGGGKGIRRVDTTEEFPGLFRQVQAEVPGSPIFVMK
LARGARHLEVQLLADQYGNAISL
Pmac ACC protein Pmac maker-sc
..........D..N........E..............................A...SAM.......I.........
...............M.......
Gleg ACC protein augustus_mask
.............KK...ST..EC...A........V............K.ENA..L.V........I......I..
..KC...I....V..N.......
Lclav ACC protein scf718000516
.............KK...STI.EC.T.A........V............K.ENA..L.A......T.I......I..
```

..KC...........N.......
Abil ACC protein Abil maker-sc
..........D.......HTA...QQ.AQ....................K..NP.D.AAS.................
..KC..................I

610 620 630 640
650 660 670 680 690 700

....|....|....|....|....|....|....|....|....|....|....|....|....|....|....|..
..|....|....|....|....|
Dmel ACC protein transcript va
FGRDCSIQRRHQKIIEEAPAIVAQPEVFEDMEKAAVRLAKMVGYVSAGTVEYLYDPEGRYFFLELNPRLQVEHPCTE
MVADVNLPAAQLQIGMGIPLYRL
Pmac ACC protein Pmac maker-sc
....A..K..................
.........C.......V..F..
Gleg ACC protein augustus_mask
....................VI.K.D...E.........................TS...Y................
.IS...........A..L..HHI
Lclav ACC protein scf718000516
....................VI.K..I..E..........................S...Y................
..S...........A..L..HHI
Abil ACC protein Abil maker-sc
....................VI..QS.............................TS.H.Y................
..S...........A..LS.HKI

710 720 730 740
750 760 770 780 790 800

....|....|....|....|....|....|....|....|....|....|....|....|....|....|....|..
..|....|....|....|....|
Dmel ACC protein transcript va
KDIRLLYGESPWGSSVIDFENPPNKPRPSGHVIAARITSENPDEGFKPSSGTVQELNFRSSKNVWGYFSVAASGGLH
EFADSQFGHCFSWGENRQQAREN
Pmac ACC protein Pmac maker-sc
.........N...T.I...
....
Gleg ACC protein augustus_mask
.............D.Q...DQ.RH..Q.W...G......
...............D.H.....
Lclav ACC protein scf718000516
.............D.A...DQ.RH..Q.W...G......
...............D.......
Abil ACC protein Abil maker-sc
.........T...D.....DQ.KH..Q.W..
.................E.....

810 820 830 840
850 860 870 880 890 900

....|....|....|....|....|....|....|....|....|....|....|....|....|....|....|..
..|....|....|....|....|
Dmel ACC protein transcript va
LVIALKELSIRGDFRTTVEYLITLLETNRFLDNSIDTAWLDALIAERVQSEKPDILLGVMCGSLHIADRQITESFSS
FQTSLEKGQIQAANTLTNVVDVE
Pmac ACC protein Pmac maker-sc
....A........S.A...

...................I...
Gleg ACC protein augustus_mask
...........................ES.QQ.N.......L......R.D...V..A.T..A......T..AA.TG
...A........S.D.E.I....
Lclav ACC protein scf718000516
..V........................EC.QQ.C.......V......R.D...V..A.T..A......T..AA..G
...A........S.D.N..M...
Abil ACC protein Abil maker-sc
...........................KS.Q..T.......I..S..M.......M...I..A.....KT.STA.NE
..I...R....GS...NHTM...

910 920 930 940
950 960 970 980 990 1000

....|....|....|....|....|....|....|....|....|....|....|....|....|....|....|..
..|....|....|....|....|
Dmel ACC protein transcript va
LINDGIRYKVQAAKSGANSYFLLMNSSFKEIEVHRLSDGGLLISLEGASYTTYMKEEVDRYRIVIGNQTCVFEKEND
PSLLRSPSAGKLINMI-------
Pmac ACC protein Pmac maker-sc
...G.N............T......N....V.........M.M.F................................
..............LL-------
Gleg ACC protein augustus_mask
.....YK..I......P.....V..G.Y..V...........L..D...V....R........I......I...D..
.............SFL-------
Lclav ACC protein scf718000516
.....YK.....T...L.T...V..N.Y..VDI.........L..D...F....R.................D.D..
..............FLVEDGGHV
Abil ACC protein Abil maker-sc
.....HK.R...T.....T...V..G......L.......I.L.VD...F....................I.D....
.T...........GFL-------

1010 1020 1030 1040
1050 1060 1070 1080 1090 1100

....|....|....|....|....|....|....|....|....|....|....|....|....|....|....|..
..|....|....|....|....|
Dmel ACC protein transcript va ------
VEDGAHVSKGQAYAEIEVMKMVMTLTSQEAGTVTFVRRPGAVLDAGSLLGHLELDDPSLVTKAQPFKGQFLQPE--
NAPVPEKLNRVHNTYKSI
Pmac ACC protein Pmac maker-sc ------
.......N...................L.....................................Y....P...--
.P.L.........I....
Gleg ACC protein augustus_mask ------
....G..DA............I..V..S...S.FY.K......E..T.IA.............SEYT...PPAA--
APAI.....HL.TK.RTA
Lclav ACC protein scf718000516
DAGQAS....G..DA...............V.AG...SIFY.K......E..T.IA..............EYL....
A.V--TPA......HL.AK.RAA
Abil ACC protein Abil maker-sc ------
....G..YR.................AG...S.SY.K.A........IIAT.....A.......LYTSP.PDLDVSH
PLAS....HI..S....

1110 1120 1130 1140
1150 1160 1170 1180 1190 1200

....|....|....|....|....|....|....|....|....|....|....|....|....|....|....|..

..|....|....|....|....|
Dmel ACC protein transcript va
LENTLAGYCLPEPFNAQRLRDIIEKFMQSLRDPSLPLLELQEVIASISGRIPISVEKKIRKLMTLYERNITSVLAQF
PSQQIASVIDSHAATLQKRADRD
Pmac ACC protein Pmac maker-sc
.....................V...
...................T...
Gleg ACC protein augustus_mask
...........D.YHLP....L.....N.................T.................S.............
......A...G.....S..S...
Lclav ACC protein scf718000516
.......F...D.YHLP...EL.....N.................T...............................
......A...G.....S......
Abil ACC protein Abil maker-sc
.....Q.F...D.Y..K...EV.....A........................PA.........S.............
..............SM....E..

1210 1220 1230 1240
1250 1260 1270 1280 1290 1300

....|....|....|....|....|....|....|....|....|....|....|....|....|....|....|..
..|....|....|....|....|
Dmel ACC protein transcript va
VFFLTTQSIVQLVQRYRNGIRGRMKAAVHELLRQYYDVESQFQYGHYDKCVGLVREHNKDDMQTVVNTIFSHSQVAK
KNLLVTLLIDHLWANEPGLTDEL
Pmac ACC protein Pmac maker-sc
N......G...............................C...H............R....................
....
Gleg ACC protein augustus_mask
.......A.................T..........T......Q.......SALI.QY...VA..TGM....N..T.
..V...M................
Lclav ACC protein scf718000516
.......T...........T.....T......K...N......Q.......SALIDQF...KTM.TS........T.
..V...M......T.........
Abil ACC protein Abil maker-sc
N...A.DG.....................D......N......L.......SAL.DKH....SM.TQI.........
..M...M......S.........

1310 1320 1330 1340
1350 1360 1370 1380 1390 1400

....|....|....|....|....|....|....|....|....|....|....|....|....|....|....|..
..|....|....|....|....|
Dmel ACC protein transcript va
ANTLSELTSLNRAEHSRVALRSRQVLIAAHQPAYELRHNQMESIFLSAVDMYGHDFHPENLQRLILSETSIFDILHD
FFYHSNRAVCNAALEVYVRRAYT
Pmac ACC protein Pmac maker-sc
....
....
Gleg ACC protein augustus_mask
SS..T.......T........A..K..............
......................V
Lclav ACC protein scf718000516
.S..T.......T........A..K......C.......
.......V..............I
Abil ACC protein Abil maker-sc
.A..N.......S........A................................E.......K..V...........

....T.................

1410 1420 1430 1440
1450 1460 1470 1480 1490 1500

....|....|....|....|....|....|....|....|....|....|....|....|....|....|....|..
..|....|....|....|....|
Dmel ACC protein transcript va
SYELTCLQHLELSGGLPLVHFQFLLPTAHPNRLFSRMSSPDGLDQAAAESLGNSFVRTGAIAAFDSFEHFEMYSDEI
LDLLEDFVSPAMVNAKVLEAVEA
Pmac ACC protein Pmac maker-sc ..D.............I...............L..A.-
EAATE.GTDN..T.Y....CM.........D............LA.ST..S.........
Gleg ACC protein augustus_mask .............EI.........NN....----------
QN.STVNH------....M...QDL.Q.SQ.A..V......LS..SS.S..I.....
Lclav ACC protein scf718000516 .............EI.......M..NN....----------
QN.SLVNH------....M...QDL.Q.NQ....V......LS..NS.S........
Abil ACC protein Abil maker-sc ..DI........AEV..I.......PS.....VTLD.I---
.EETEPAKVFD..Q...CM...E..QQ..S.A...F..I...AN..TIS..D.NML.S

1510 1520 1530 1540
1550 1560 1570 1580 1590 1600

....|....|....|....|....|....|....|....|....|....|....|....|....|....|....|..
..|....|....|....|....|
Dmel ACC protein transcript va ADSISDSR-HSTSINVSLSDPVTRANAAEEA-
KSTEPIHIVSVAVRETGELDDLQMAQIFGNYCQEHNEELFQRRIRRITFAALKKRQFPKFFTFRARDK
Pmac ACC protein Pmac maker-sc ....G.G.-L...........IS........-
........I.........M..V..........KQ.RD.........................Y....N
Gleg ACC protein augustus_mask .G--.E..-........I.-
TAEPSTTI.RGERPS..V..L.I..Q.IDNQ..TAL.RM..DW.ANNKD..IS.G...V......R..........Q
..G
Lclav ACC protein scf718000516 VG--.E..-
.........TT.EGNAQN.SGDDPA..F..L.I..IDK.NQ..AT..RV..DW.ALNKD..IA.GV..V..L....
.....L....Q..G
Abil ACC protein Abil maker-sc G---G...TN..........IDGQ.QITEDSN-
.VC.....LHIG.KDK.DE..ST.SR...SF.ER.RQD.ET.G..........HK.......Y....G

1610 1620 1630 1640
1650 1660 1670 1680 1690 1700

....|....|....|....|....|....|....|....|....|....|....|....|....|....|....|..
..|....|....|....|....|
Dmel ACC protein transcript va
FTEDRIYRHLEPASAFHLELNRMKTYDLEALPTANQKMHLYLGKAKVSKGQEVTDYRFFIRSIIRHSDLITKEASFE
YLQNEGERVLLEAMDELEVAFSH
Pmac ACC protein Pmac maker-sc
YE..........C.YQ.........................R..........F....................
....
Gleg ACC protein augustus_mask
.V.........GC..Q......R.........S.........Q...A...Q.......................D
..H...................
Lclav ACC protein scf718000516
.F...V......GC...I.....R.........S.........Q...A...Q.......................D
..H...................
Abil ACC protein Abil maker-sc
.K..........C..Q......R..N......S.............AP.H........................
....

1710   1720   1730   1740
1750   1760   1770   1780   1790   1800

....|....|....|....|....|....|....|....|....|....|....|....|....|....|....|..
..|....|....|....|....|
Dmel ACC protein transcript va
PHAKRTDCNHIFLNFVPTVIMDPAKIEESVTKMIMRYGPRLWKLRVLQAELKMVIRQSPQSPTQAVRLCIANDSGYF
LDISMYTEQTEPETGIIKFKAYG
Pmac ACC protein Pmac maker-sc
.F...L...N..A...S............
........H.DK...V...M...
Gleg ACC protein augustus_mask
.L....E........A.N......R......S.VL...........R...I..T..PA.GK..TNI..........S
I.LHL...A.D.K....R.ESFP
Lclav ACC protein scf718000516
.L......................R......S.VL...Q.......R...I..T..PA.GK..SN.....S.....S
I.LHL...AIDQK....R.ES.-
Abil ACC protein Abil maker-sc
.QSR...........I.......S....A..S.V...................T..S..T...TT...........Y
...N....VVNVD....R.E...

1810   1820   1830   1840
1850   1860   1870   1880   1890   1900

....|....|....|....|....|....|....|....|....|....|....|....|....|....|....|..
..|....|....|....|....|
Dmel ACC protein transcript va E--------
KQGSLHGHPISTPYMTKDFLQQKRFQAQSNGTTYVYDVPDMFRQMTERHWREFSKARPT-
VDIRTPDKILIECKELVLEGDN---LVEMQR
Pmac ACC protein Pmac maker-sc .--------
.......L.............................I..........L.K.Y.M....-
....I.E......V....D...---....K.
Gleg ACC protein augustus_mask
SQNPNNPNPRI.PM..L......L...Y..A.......A.......L......QL.KT.AKYIDE.SAIEP.TM.NP
VM-DSV...V..E.---...LK.
Lclav ACC protein scf718000516 SGSANNSN-
RP.PM..L......L...Y..A.......S.......L......QV.KS.K..IDE..S-EV.TI.NPLI-
.IV....D.-D---...LK.
Abil ACC protein Abil maker-sc T--------
...P...L......LA..Y.........QS.......Y.......VDLL.KQY.QE.MN-EVVVI.E.VM-
D.I....DPE.ESR...QK.

1910   1920   1930   1940
1950   1960   1970   1980   1990   2000

....|....|....|....|....|....|....|....|....|....|....|....|....|....|....|..
..|....|....|....|....|
Dmel ACC protein transcript va
LPGENNCGMVAWRIVLATPEYPNGREIIVIANDLTYLIGSFGIKEDVLFAKASQLARQLKVPRIYISVNSGARIGLA
EEVKAMFKIAWEDPEEPDKGFKY
Pmac ACC protein Pmac maker-sc
.....T........I.......E...M........FF......P..IV.H.......SR..................
........V..............
Gleg ACC protein augustus_mask
.....DV......FT.Y...C.T..DV.L.G..I.HM......PR..I..YR..ER....I....FAA.........
.....L.......EM..E.....

Lclav ACC protein scf718000516
.....DV......LT.Y...C.T..D..L......H.......P..I..F...ER....GI..V.F.A......A..
.....L.R.....EA..E.....
Abil ACC protein Abil maker-sc
V.....V......LT.Y.....A..I.......I.F.M...APR..KV.GL..E...N........AA.........
.....LY....D..N...R..R.

2010 2020 2030 2040
2050 2060 2070 2080 2090 2100

....|....|....|....|....|....|....|....|....|....|....|....|....|....|....|....|..
..|....|....|....|....|
Dmel ACC protein transcript va LYLSTEDYAQVANLNSVRAILIED-
EGEQRYKITDIIGKDDGLGVENLRYAGLIAGETSQAYEEIVTIAMVTCRTIGIGSYVVRLGQRVIQIDNSHIILT
Pmac ACC protein Pmac maker-sc ...T....SR..K...........-
...P...........E.....................D.....S............L..........E.......
Gleg ACC protein augustus_mask
I..TPD...RL.P....K.S....PA..S...........Y.I.....K...M......K..D.V...SI.S..A..
....L..........E.......
Lclav ACC protein scf718000516 I..TPD...RLSP....K.S....-
G..S..R................K..........K....V...SV.S..A.................E.......
Abil ACC protein Abil maker-sc ...TP....K.SAW.....V....-
...S.............F.....Q..............D....S..S..A....A.L..................

2110 2120 2130 2140
2150 2160 2170 2180 2190 2200

....|....|....|....|....|....|....|....|....|....|....|....|....|....|....|....|..
..|....|....|....|....|
Dmel ACC protein transcript va
GYAALNKLLGRKVYASNNQLGGTQIMFNNGVTHKTEAIDLDGVYTILDWLSYIPAYIGCDLPIVLP-
NDRIERPVDFMPTKSPYDPRWMLGGRVNPVNAN
Pmac ACC protein Pmac maker-sc
......................V...Y..........L..........................E.-
....D..................A.....G..S
Gleg ACC protein augustus_mask
.NR...AV...E..........V...H....S.S.DVR.....A.A.K....V.KAK.AP...LP.LL.P...E.MY
T.........F..D...S.SDP.
Lclav ACC protein scf718000516
..R...TV...E..........I...H...IS.AI.PR.....E.V.R....M.KSK.AP...IES-
I.P.D.EIG.V...A........E.K--Q..DH
Abil ACC protein Abil maker-sc
..S........E..........I...Y....S....PR....I....K......KDKLSGV..LP.-
T.PYT.EIGY....A........A..Q..NSPA

2210 2220 2230 2240
2250 2260 2270 2280 2290 2300

....|....|....|....|....|....|....|....|....|....|....|....|....|....|....|....|..
..|....|....|....|....|
Dmel ACC protein transcript va
DWENGFFDRDSWSEIMASWAKTVVTGRARLGGVPVGVIAVETRTVEVEMPADPANLDSEAKTLQQAGQVWYPDSSYK
TAQAIKDFGREELPLIVFANWRG
Pmac ACC protein Pmac maker-sc
E................P..............I..
.S.............MI......
Gleg ACC protein augustus_mask

V..S.....N..Q...KP..Q...........I.C..........LHL............IS.........A..
.........H.....FI......
Lclav ACC protein scf718000516
T..S.....G..Q...KP..Q...........I.C.I........LHL............VS......F...A..
........NK.....FI......
Abil ACC protein Abil maker-sc
E..A....K.......QP..Q...........I............LK.............VS......F...A..
.....Q...K.D...FI......

2310 2320 2330 2340
2350 2360 2370 2380 2390 2400

....|....|....|....|....|....|....|....|....|....|....|....|....|....|....|..
..|....|....|....|....|
Dmel ACC protein transcript va
FSGGMKDMYEQIVKFGAYIVDGLREYKKPVLIYLPPNAELRGGAWAVLDSLINPRYMETYADPEARGGVLEPEGIVE
IKYKEKDLVKTIHRLDPTTIALK
Pmac ACC protein Pmac maker-sc
............................I...........................S.................
................A..----
Gleg ACC protein augustus_mask
...........M............TR.I.V.I...G.........V.PT...D...MF..NTS......DA...
..F.TR.TL..M..V.HIIQK..
Lclav ACC protein scf718000516
...........M............TR.IVV.I..YG.........V.PM....H..MF..HTS...........
..FRN..I...M..N.SVIHN..
Abil ACC protein Abil maker-sc
..........V...........K.R..II..I...G.........V.PF..S....M.........I.......
...RK...L..M..I.A.LMQ.D

2410 2420 2430 2440
2450 2460 2470 2480 2490 2500

....|....|....|....|....|....|....|....|....|....|....|....|....|....|....|..
..|....|....|....|....|
Dmel ACC protein transcript va KELDEANASGDKVRAAQ-------------------
VDEKIKARIAVLMHVYHTVAVHFADLHDTPERMLEKECISEIVPWRDSRRWLYWRLRRLLLEDA
Pmac ACC protein Pmac maker-sc ---

Gleg ACC protein augustus_mask EQ.SNTSSP--------EERSQ----------------
IEA...E.EQQ.EPM.RQI...............INA.ND.....R..KL.......R.F.EE
Lclav ACC protein scf718000516 EK.ASCGSA--------EERAS----------------
.ERE.HD.ECH.ESM..Q................NT.Q...A..SA..I.......R....R
Abil ACC protein Abil maker-sc
EK.KML..ANVPIEILERRGSVTQTPERKKTPEIIA.EKE.VE.ENY.LPM..Q...N...........H..GT.LD
.....K..TI..........Q.R

2510 2520 2530 2540
2550 2560 2570 2580 2590 2600

....|....|....|....|....|....|....|....|....|....|....|....|....|....|....|..
..|....|....|....|....|
Dmel ACC protein transcript va
YIKKILRAQDNLSVGQAKQMLRRWLVEEKGATEAYLWDKNEEMVSWYEEQINAE---
SIVSRNVNSVRRDAIISTISKMLEDCPDVALDAVVGLCQGLTP
Pmac ACC protein Pmac maker-sc ---
--------------------------------------K..SF-------------

```
Gleg ACC protein augustus_mask
IRSEV.ST.PG.DIR.VGA.....FI.D..T..S....QD.TAAR.L.N.L.D.--
N.V....IAC.KK.TVVTR.KES..AY.E.R.N.MLEIVHR.HS
Lclav ACC protein scf718000516
IRSE.IST.PG.D.R.VDA.....F..D.....S....QD.VVAT.L.A.CEN.--
S.V.M..ISC.KN.S.VTRVKEA..V..E.RF...LEIVNR.Q.
Abil ACC protein Abil maker-sc
V.TQL.ETNS..GI..GEA.....F......S.G.K..N..AV.E.L.K.MSV.NEN.ML...LHA.KK..L.QK.K
NSI.....L......EIL.K.ND

                                       2610      2620
                                ....|....|....|....|....|...
Dmel ACC protein transcript va  VNRGVVVRTLAQMQLNEETSNSNQG---
Pmac ACC protein Pmac maker-sc  ----.QY.CF------------------
Gleg ACC protein augustus_mask  TE.AELL...S.IEASGQEHHNDSNVSS
Lclav ACC protein scf718000516  AEIAELQ.....LESTSQENHNDSSASS
Abil ACC protein Abil maker-sc  NQKAE.I...S.V.PET.S---------
```

Supplementary text 2: Fatty acid synthase (FAS) amino acid sequence alignment for *D. melanogaster*, *P. maculata*, *L. clavipes*, *G. legneri* and *A. bilineata*:

```
                                        10        20        30        40
50        60        70        80        90        100

....|....|....|....|....|....|....|....|....|....|....|....|....|....|..
..|....|....|....|....|
Dmel FAS transcript protein    MPARF-------
AEEVITAEPAQRAAPQLDLGGGHYVPRQQHLNDEIAITGFSGRLPESSTIEEFKQNLFDGVDMVNDDPRRWERGLYG
LPDRIGKLKDS
Lclav FAS transcript protein    .....ESVNAPIVRDQ.IPNGT-----------------
TYSID.D.V............N......K....I.L.T..E...PS..H...T.T....--
Gleg protein FAS                ...Q.ESMNTPIVR.PVVTNGSR----------------
SFSMD.D.V.S.........EN....QK...E.I.L.T..E...PS..H...T.T....--
Abil protein FAS                ....-----VPEMR-----NGE-----------------
CHQA...VV...L.......Q.......Q.....L.T..E...TA..H...T.T..I.--
Pmac protein FAS                .....-------VDG..NENGGRN.PHD.QERF.LHSSGPMTVS.-
..............N.....K...E..............K........L..I..E


                                       110       120       130       140
150       160       170       180       190       200

....|....|....|....|....|....|....|....|....|....|....|....|....|....|..
..|....|....|....|....|
Dmel FAS transcript protein
DLENFDQQFFGVHQKQAECMDPLLRMLLELTHEAIIDAGLNPSDLRGSRTGVYIGVSNSETEQHWCSDADRVNGYGL
TGCARAMFANRISFTFDFKGPSY
Lclav FAS transcript protein
.IAS..AT.....A...HV...Q...............I...EI...K...F....D..SDEF.TA.P.M......
...C....P....Y....T...F
Gleg protein FAS
.IAS..AT.....A...NV...Q..LM..A.....V...F..T.V..T....F....S..SDDF.TRSPET......
...C....P....Y....N....
Abil protein FAS
.ITH..AT.....A...HL...Q...........L....I...EF...N...FV...E..SSEQ.TR.P.AI...S.
...C....P....Y...L.....
Pmac protein FAS
...H......S...........QM.....C.Y.....S.V...EV............A.....Y.TA.Q........
...........V.Y.........


                                       210       220       230       240
250       260       270       280       290       300

....|....|....|....|....|....|....|....|....|....|....|....|....|....|..
..|....|....|....|....|
Dmel FAS transcript protein
SIDTACSSSLYALEQAFSDMREGKVDNALVAGAGLILKPTMSLQFKRLNMLSPDGSCKAFDESGNGYVRSDGCVVLL
LQRTSAARRVYASILNVRTNTDG
Lclav FAS transcript protein
AV.......M..MH..VAA..T.QC.S.I.G.VN.V....S....H......M..A.....A........EAA..IF
..KARNS.....TVV.SK.....
Gleg protein FAS
A.........F.MH..IAA..S.EC.A.I.G.VN.C....A....HK.S...ME.A.....A........EAV..VF
..KSKD......TVV.SK..V..
```

Abil protein FAS
A........MF..Q..YAAIKS.QC.S.I.G.VN.L....N....H..G...AE.K.....A..S....AEAA..IV
..KA........T..GAK.....
Pmac protein FAS
.V.......F...........D..C.S.V...L.V....................M...................IF
..K..QSK....TV.........

310 320 330 340
350 360 370 380 390 400

....|....|....|....|....|....|....|....|....|....|....|....|....|....|....|..
..|....|....|....|....|
Dmel FAS transcript protein
FKEQGITYPIGKMQNRLIRETYEEIGLNPADVVYVEAHGTGTKVGDPQEVNSITDFFCKDRTTPLLIGSVKSNMGHS
EPASGVCSVAKILIAMEEGVIPG
Lclav FAS transcript protein
N.VE...F.S.A...K.M..V.A.V.VD.V.......................A.L.....KK......I.......
.....L..I..M.L...A.....
Gleg protein FAS
Y.DL.....S.S...K.M..I.D.C.V..S..T....................A.L.....KN...L..........
.......AI..L...L.T.M..P
Abil protein FAS
S......F.S.QV.....N.V.ADS.IE.N.......................A.....N.K....L..........
.....L..I..M.....S.Q..A
Pmac protein FAS
Y........D.R.............N.D.NE.A..........................N.................
........I..V..........A

410 420 430 440
450 460 470 480 490 500

....|....|....|....|....|....|....|....|....|....|....|....|....|....|....|..
..|....|....|....|....|
Dmel FAS transcript protein
NLHYNKPNPDLYGLVDGRLKVVDRNLPWNGGIIGLNSFGFGGANAHVILKSNPKPKALTPK--
DGALKVVLASGRTFEAVEQLLESASTNADDDEYLQLI
Lclav FAS transcript protein
...FKS..K.IPA.S....Q....SM.....LVAI...........LV.R......IA-
.VLDVNVP.I.PV....DD..NLF.DRIKEHEK...FTSMV
Gleg protein FAS
...FQN..K.IPA.S...IQ..TQPTAYK.NLMAV...........ILVRGHS...LS-
.VMDR.VP.L.AV....N...NVM.DKIKEHHR...FIA..
Abil protein FAS
...FKN..T.IPA.C...I...AT.EK.T...V.V..............R......ENW.V--
EQLPRL.VV....ED..NHF.DKIKEQSH.E.FYAML
Pmac protein FAS
....KN........M........K....E.....I.....................TI..T--
V.PP.M.VC.....D..QE...D.TSHR......A..

510 520 530 540
550 560 570 580 590 600

....|....|....|....|....|....|....|....|....|....|....|....|....|....|....|..
..|....|....|....|....|
Dmel FAS transcript protein
NEIHSKAIPNHFFRGYGVVSSKGTHQREVIESNDDKRPIWYIYSGMGSQWASMAKDLMKIEAFAKTIQRCADVLKPE
GVDLIDVLTRSTDKSFENILNSF

```
Lclav FAS transcript protein   QDL.ANN.TG.GY..FQILGDVN.--
..IDQVGSE.......F.......SG.GRA.FC.DT.QSA.R...EA.....I...NLILNG.EE..Q.VV...
Gleg protein FAS               H...N.N..G.N....QILGGED.--
..IL.NHSA.....FVF.......PG.G.E.LHLDV.NRSLR...EA.RS.....M.IIQNG.NET....I...
Abil protein FAS               .N..A.N.TG.NY..FA.LGDNEI--
.D.SMVGNE.K...FVF.......PG..R.....DL.QQ..K.A.QA.N.Y....E.I.LN..EETLT.VR...
Pmac protein FAS
.D....N..L.YY...C.MDT..SL....L.F..EN..V.................QF.V..NS.H...KA.R..
.I..V........L..D......

                                       610       620       630       640
650       660       670       680       690       700

....|....|....|....|....|....|....|....|....|....|....|....|....|....|....|..
..|....|....|....|....|
Dmel FAS transcript protein
ISIAAMQVALTDLLSSLGIHPDGIVGHSVGELGCAYADGCFTPEQTVLAAYWRGKSILDTQLAKGKMAAVGLSWEDA
HSRVPSDCFPVCHNSEDNCTISG
Lclav FAS transcript protein
V....I.IG.V.V.NLI..Q....I...I.....G....TM..S...........T...GN.PP.A.........E.
QK.C.PEIVLA...AA.SV....
Gleg protein FAS
V....I....V...T.......................T......I....L...A.AESD.PA.C..........T
KA.C.P.VV.A...AA.SV....
Abil protein FAS
....SI.IG.LEI.K..N.E....L...I..V.......TL.L...IQL.WA..TA..ESD.PP.A......T..EC
KK.C...I.........SV....
Pmac protein FAS
.............T..N.K...................S...............Q..K.PP....SI..D..E.
.K.M.A.........A.......

                                       710       720       730       740
750       760       770       780       790       800

....|....|....|....|....|....|....|....|....|....|....|....|....|....|....|..
..|....|....|....|....|
Dmel FAS transcript protein
PEASIEALVAKLNAEGVFAKAVNSSGYAFHSKYIAEAGPKLRKSLEKIIPNAKNRTARWISTSIPESAWNTPVAKQS
SAAYHVNNLLSPVLFHEALQHVP
Lclav FAS transcript protein
.PEPLAKF.EE.KSQEI...Q.H...C........SV.....TI.......P.Q.SS....S....T....SL.QL.
.P..Y..........Q...A...
Gleg protein FAS
.TG.V.KFIEE.KK.EI.....K.N.I........S......AG....LT.P.Q.SS....S.......G..L.QL.
.P.............Q...A...
Abil protein FAS
.P...DKFT.E.TK..I...K.....F........A.......A.DT..Q.P.A.S............G..L.Q..
N...........Y....TK.I.
Pmac protein FAS
....D....Q.SS........K............D..........R.........NK..............I....
...............Y.....I.

                                       810       820       830       840
850       860       870       880       890       900

....|....|....|....|....|....|....|....|....|....|....|....|....|....|....|..
..|....|....|....|....|
```

```
Dmel FAS transcript protein
KNAISVEIAPHGLLQAILKRALGPDATNLSLVKRGHENNVEFFLTNVGKLFAAGAQPQVLTLVRPISYPVGRGTPML
NSKVGWDHTQKWLVAKF-GKETS
Lclav FAS transcript protein
D...VI.....C......R.SFPSTV..IG.H..D.SD.LA.L...I...YV.....ILSK.YP.VT..........
..MIL...STQ.S..D.S..SGN
Gleg protein FAS
ED..AI.....C......R.S.PKTV..I..H..D.TD.MNYL.S.....YC......ISK.YP..NF........I
..MIK...SVQ.G..NYAQNSAR
Abil protein FAS
D...VI..............G.SK.CA.I........D.AR.L.S.I.RI.N..G..NIAN.YH.V.F.........
A.MIE...STE.S..NYCD.NDR
Pmac protein FAS
....AI....T.................V.............L.M.I...Y....K.....MFK.............
........S...N.P.Y-....T

                                        910       920       930       940
950       960       970       980        990       1000

....|....|....|....|....|....|....|....|....|....|....|....|....|....|....|..
..|....|....|....|....|
Dmel FAS transcript protein
SGETIVEVDLSKEDDAFLAGHTIDGRILFPATGYMTLAWQTFAKMQGSEFHKTPVVMENLVFHRATILNKNAVVKFG
INFFDGTGAFEICESGSLAVSGK
Lclav FAS transcript protein
..QSV..F.....S.SYI...C............L.IV.K....LR..SYE.....F.DVQ.L....MP.EGS...I
..I.E.......S....ST.I..
Gleg protein FAS
...S...I...T.S..Y.................L.IV......LHNE..NRM.IIL..VQ......MP.EGK...L
..I.E...D.......I..T..
Abil protein FAS
..QFVIDI.....EHKY.V...............L..V.K.....RNQD.EQL..II.DVK.M....MP.EGS...L
..I.E.S.E.......V.....
Pmac protein FAS
....VI.IN.G..E.S.F......................M.....K.M.YQ.C..I...I..........EG.....
L.......N.....G........

                                        1010      1020      1030      1040
1050      1060      1070      1080       1090      1100

....|....|....|....|....|....|....|....|....|....|....|....|....|....|....|..
..|....|....|....|....|
Dmel FAS transcript protein    ITIPESIDNEELPLEEQTPSAVAKE-----
LGTNDVYKELRLRGYDYGGIFRGIVRSDTVASTGKLQWVDNWISFMDTMLQFSILSKNLRELYLPTRIER
Lclav FAS transcript protein   .R.S.D.EKDQ.N.---
PIPVTCN.PDLLE.K......D........S...Q..KS..NR.I..N.A.NND...Y.......A..G..T.D.F.
...LQY
Gleg protein FAS               .RRA.Y.E..Q.N.---
PIPVLR..ENILD.N...I..D........S...Q..KSA.NRGII...T.SND..............G..T.D.F.
...LQY
Abil protein FAS               .YVA.EPEKQF.T.---
PKHTLI..KDILD.N.P.I...........D......HSA.NYGLV...K.EQ.....I......N.....T.....
...LQ.
Pmac protein FAS               .S...D.EM.....DALPA.TLG..-----
.N..............S.......K.....N..Q...AE..V...........................K

                                        1110      1120      1130      1140
```

1150 1160 1170 1180 1190 1200

....|....|....|....|....|....|....|....|....|....|....|....|....|....|....|..
..|....|....|....|....|
Dmel FAS transcript protein
AVINPAKHFELLSALTKEEQVETGLPVQWYSDINVIKSAGVELRGLKANLAQRRPGTQAPPTLERYQFVPNINTTDL
NENSEKARLHALDVAIQVIIENS
Lclav FAS transcript protein .A...ER.IQ.VEK.QEN.----
NI..FH..NVGIV..G......M.SSI.P..QQ...D.K..K.S.I.YE..QA.V.DP..SK....TSLL..VR..I
Gleg protein FAS .A....L.MH.V.G.KSD.----
....YS..N.GIL..G.I....M..S..P..QQA....KH...T...YETNNA.V.DPQ..KV..MC.LF.I.C..M
Abil protein FAS .I...VE.IR-----NAK.----
HVT.SM.R..D....G.........S..P..QQS.SA....Q...L.YL.MNQVVDEQI-----T.T..S.IAL...
Pmac protein FAS
........LATV.K.SE.YLTLN....YM.G.......G...M.....S..SK...S.N.......T.L..V.YAE.
H.....S..Q..T..L.T.M...

1210 1220 1230 1240
1250 1260 1270 1280 1290 1300

....|....|....|....|....|....|....|....|....|....|....|....|....|....|....|..
..|....|....|....|....|
Dmel FAS transcript protein
SGAVKLKGVELANGRNPDVLVANRLLQIIEGEPVLTGDVAVVTSNNNEETITAALG-
DSGVRVVSKDVLKEPVEQNCHFVFGIDVLSRPDTKTLENSIAS
Lclav FAS transcript protein G.-I.I.AI.TTME...EA.LTPIV.D.LLS..M.AV.LKLA.T--
VPDNY.PIME-QCN.KTTVV.IHSS..G.DMQLIITA.IMNNQMIAAVK.LE..
Gleg protein FAS GS-M...II.V.GE.SAES.L.PTVMDVLYS..LMSV.IQIA.T--
TP..YN..ME-QYN.KT.VR..NSN.AG.DL.T.IAP....NKNVNM.K.IA..
Abil protein FAS G..L.M.V...QGSK.IEQ.LIPKVQG.L.CQ.M..VE-SILV.--
Q.NID....E-EKSIK.SR..PSADAF...A...LMS...AYNKSEV.T.AFK.
Pmac protein FAS
Q..I.I.............M.VK.........TI.A....A.Y.......AST..T.....II..NI.E.......
..LYAL........MI....K.T

1310 1320 1330 1340
1350 1360 1370 1380 1390 1400

....|....|....|....|....|....|....|....|....|....|....|....|....|....|....|..
..|....|....|....|....|
Dmel FAS transcript protein
IRENGFLILEETLPTYTKTGRALLTKFGFVAVQEQSLGATRVLVLARKAVDLKTRKSVVVVATEQNFNWVDDLKAAL
ATAATEEQYVYVVCQGEELFGAV
Lclav FAS transcript protein .KPG..ILT..ATEI----DESI.KGSSLIVIGK.VVPG-
KSYI.LK.K-EEMD-VPL.IKV..K..S..N...V..KKSE..G.K.L..S....AL.L.
Gleg protein FAS LKNG..A.....GAV----DMK..NGT.LLYAGK.ISAG-
KTYI.LK.R-ED.K-EPIIIQI..R..S.LEGV....KKSE..G.E.LL.S.....L.L.
Abil protein FAS LKPG..VLF..SSNF---SDYS.F.SQELEI.YQ.RTPM-
KIYI....Q.QVAQ-DAIIIEV..NTYS..EPI.Q.MKESE.NNRKI.LIV.....S.L.
Pmac protein FAS
.KD.....F..STTS.G.SS.D..H.Y.LIV.T..VI.GS....M...P....Q.DA...HV..A..D.LE...E..
.K..EI.R..............

1410 1420 1430 1440
1450 1460 1470 1480 1490 1500

....|....|....|....|....|....|....|....|....|....|....|....|....|....|....|..

..|....|....|....|....|
Dmel FAS transcript protein
GLMTCIKNENGGKLARLVFVQDAKAEKFSLTSTLYRQQLEKDLISNVLKNGAWGTFRHLKLETQQ--
ATLQVEHAYVNALVKGDLASLKWIEAAQADTAA
Lclav FAS transcript protein
.....VRQ.A..MNV.YF.I..VN.SA...DDAF.AK.FD.QVMA....G.Q..SY...R.DK.SDIPS.......I
...TR...S..R...GPLCYYEP
Gleg protein FAS
.F....RR.P..MN..Y..I..KN.P..G..TPF.AD..S.Q.A.....G.Q..SY...R.DQ.NDASS.......I
.T..R...S..R...GPLSYYQP
Abil protein FAS
.MVN.L.Q.P..VNM.A.LI..T...T.N.S.KFFVD..Q...VH......I..N....S.SMEK--
S........I.T.TR..........GPLGYYNN
Pmac protein FAS
.F.N...........M..I..KN......NK..AE..S....N.....SV..........DV--
.............T...........P.L-CS.

1510 1520 1530 1540
1550 1560 1570 1580 1590 1600

....|....|....|....|....|....|....|....|....|....|....|....|....|....|....|..
..|....|....|....|....|
Dmel FAS transcript protein
TVDKNLETCTVYYAPINFRDVMLTSGKLAADALPGDLAEQDCVLGLEFAGRDTQGRRVMAMVPAKSLATTCVASKRM
MWQIPEKWTMEEASTVPCVYSTV
Lclav FAS transcript protein
EKFVGQ.F.D.....L....I..AT...PP.......S...I.........SS.....G..A.RG....LL.DPGF
..EV.D...L...A.I.V..A.S
Gleg protein FAS
EKYP.T.M.S.....L....I..AT...PP.......G...I.....S....K....IG.IA.RG....VL.DPGF
L.EV.D...L...A.I.V..A.S
Abil protein FAS DD-
P.A.L.S.....L....I..AT...PP.......G...I.....S...SR.....G..A.......VL.DPGFL.E
V....SL...A.I.V..G.S
Pmac protein FAS
LL.Q...L...............S....S.........Q............SK...I................N.
..E..DN............A..

1610 1620 1630 1640
1650 1660 1670 1680 1690 1700

....|....|....|....|....|....|....|....|....|....|....|....|....|....|....|..
..|....|....|....|....|
Dmel FAS transcript protein
YYALVVRGQMKKGEKILIHAGSGGVGQAAISVALAHGLTVFTTVGSKEKREFLLKRFPKLQERNIGNSRDTSFEQLV
LRETKGRGVDLVLNSLSEEKLQA
Lclav FAS transcript protein
........RLRP..S.............S.AI..HA.C......S........K.T..Q.TDK.............
.T..G.................
Gleg protein FAS
....F...SLRP..S......T......S.AI..H..CK.............K.T..Q.TD.............
.T..N.....V.....A......
Abil protein FAS
....I...GLRP..S..V...T......S.AI..HM.CK.....S.QA..D..K....Q.TDNQ...........I
.TQ............AG.Q...
Pmac protein FAS
.......................S...I..H................K....Q.KDSH......C....MI

M.Q.Q...........A......

1710 1720 1730 1740
1750 1760 1770 1780 1790 1800

....|....|....|....|....|....|....|....|....|....|....|....|....|....|....|..
..|....|....|....|....|

Dmel FAS transcript protein
SIRCLGLNGRFLEIGKFDLSNNSPLGMSVFLKNTSFHGILLDSVMEGEEEMQNQVVSLVAEGIKTGAVVPLPTSVFN
DQQVEQAFRFMASGKHIGKVVIK
Lclav FAS transcript protein
.V...AKD...............A....F.............ALFDTNGPEKKE..R..Y....S...R...AT..T
E..I..G.....A.......LL.
Gleg protein FAS
.V...ANG..............N...........T.......ALFDTDCPEKRE..KI.N....N...R...STI..
EN.I..G..Y..T.......LL.
Abil protein FAS
.V...ANG...C....V......A....L.....T.......ALF.SDCSEKKE.MR..S...AN...Q...ST.YG
ET.A................LL.
Pmac protein FAS
.V...D..............S...R........
EH...S...............V.

1810 1820 1830 1840
1850 1860 1870 1880 1890 1900

....|....|....|....|....|....|....|....|....|....|....|....|....|....|....|..
..|....|....|....|....|

Dmel FAS transcript protein
VRDEEAGKKALQPKPRLINAIPRTYMHPEKSYILVGGLGGFGLELTNWLVTRGARYIVLTSR-------
SGVKTGYQGLMIRRWQERGVKVVIDTSDVTT
Lclav FAS transcript protein I.....-
Q.ITR.AMKTVA.......N.D...V.........M..A..MI....KN......XXXXXXXX.IR....A.C....
R.M.ITIQ.S.C....
Gleg protein FAS I....P-
N.IVPYS.KTVP.......N.....V.........M..A..MI....K..I....-------
..IR....S.C....M.M..NIHVC.H....
Abil protein FAS I....S-
R.T.I.AIKTVT...K...DT..V..................I....KK......-------
..I.....SMC....RSQ..T.L.S.A.A.K
Pmac protein FAS
.....D.R.T.K.SS..V...............I................S...K.L..S..-------
..I.....S.............L.......S

1910 1920 1930 1940
1950 1960 1970 1980 1990 2000

....|....|....|....|....|....|....|....|....|....|....|....|....|....|....|..
..|....|....|....|....|

Dmel FAS transcript protein
AAGAKKLLENSNKLALVGGIFNLAAVLRDALIEDQTAKDFKTVADPKVTATKYLDQFSRDICTELDYFICFSSVSCG
RGNIGQTNYGLANSAMERICEQR
Lclav FAS transcript protein
EV..DS..KEA....P...............M.NLEEDH..V.TL...NG.RN..AS.KKF.P...F.V........
...M...............M...
Gleg protein FAS
LS..EE..KVC.RI.P.................NLDEGQ..A.VA..I.G..N..TA..SL.PS....VV.......

...M...............V.S.
Abil protein FAS
PE..RQ..NE.A..GPI.A...........FM.NLSEA..N..CK...D......AA...LAAN..H.V....I...
...A..S.......V......M.
Pmac protein FAS
DK.CQQ..I.A..F.................L....V...Q..CES..QG......Y..AM.S.....V........
.....................L.

2010 2020 2030 2040
2050 2060 2070 2080 2090 2100

....|....|....|....|....|....|....|....|....|....|....|....|....|....|....|..
..|....|....|....|....|
Dmel FAS transcript protein
QVSGFPGTAIQWGAIGDTGLVLENLGDNDTVIGGTLPQRMPSCLQTIDLFLQQPHPVVASMVVAEKRKSD-
QSAGVSLIATIANILGLRDTKNIQDGASL
Lclav FAS transcript protein
.AV.L..L.........V..I..TM.N.E.EV........A...M.M.S........L....L..RQ.AGDS.SQ.N
.LDAVG....IK.V.TVNMNN..
Gleg protein FAS
.GI.L..L.........V..I.DTM.N...EV........W...S.M.T........L....L...N.PTDSANQI.
.VDAV.....IK.....NVNN..
Abil protein FAS
.SV.L..L.........V..I..TM.G...EV......KIS..MA.M.I......A......L......G-
GDNQ.K.TDAV.....IK...TVPAI...
Pmac protein FAS
.A.....L.............I......N...........T.......F........L............-
..G.....SC...............S...

2110 2120 2130 2140
2150 2160 2170 2180 2190 2200

....|....|....|....|....|....|....|....|....|....|....|....|....|....|....|..
..|....|....|....|....|
Dmel FAS transcript protein
ADLGMDSLMSAEIKQTLERNFDIVLSAQEIRQLTFGALKAMDGGADVKP--
AAAAPAAAAGVPEANITSGGSSRTASPMGDGTQVVFT-TSLIPTEAIVQ
Lclav FAS transcript protein
.........GT.........Y.L........A....K.LELSS.SAEANEV.SQS..NSS--------------
LTETDP.EFLFQCSG.EIV.PKSLI.
Gleg protein FAS .........GT........G..L...P....N....K.MELSSD--
-----VSI..STS-----------------ESQPENLLFQYSSNEIV.F.PL.K
Abil protein FAS .........G..........Y.L..N.....A...AR.VELES.G-
-----.ST....S------------------DN..KNL.Q.V-DE.M..QLL.N
Pmac protein FAS
.........G..............M.P........IQ..QLS...ESSD--...S..SPV----------
RR.PSP..F.....M..S-.E.M..Q...R

2210 2220 2230 2240
2250 2260 2270 2280 2290 2300

....|....|....|....|....|....|....|....|....|....|....|....|....|....|....|..
..|....|....|....|....|
Dmel FAS transcript protein
LDTKAPANSKQSPIFFISPIEGFASALEPLAKRLEVPAYGLQYTEAVPSDSLESAAKFFIKQLRTVQPKGPYKLAGY
SFGCLLTYVMAGILEETNEVANV
Lclav FAS transcript protein .---

```
QSTSE.GE...V.HA...VV.S.KS..SE..R.VW...C.KDA.L..IPNL.TYY.QEMK..KKQ...SII.....A
CVAFE..LQ..KAG.T.EL
Gleg protein FAS                F---
DSIGIGKP...MVHA...SVAG.KL..SA.NITVF.I.C..D..L..IPEL.AHYV.LMTS..KV...R.F.....A
CVAFE..LQM.AIGHKLDL
Abil protein FAS
MNNVDSTE..KT...ILH....AVTI.KKF.QEIQA.V..I.C...A.LS.INDL..YY.E.IK.M......T.I..
...ACVGFE.GIQ..AM..KVKL
Pmac protein FAS
.QSA...E..KR.L.VV.......D..KQ..S..DC.V....C.AEANLE.IDTL.D.YL..I....AR...AI...
.Y.A.VG..IVLH..KMK.N.RL

                                       2310      2320      2330      2340
2350      2360      2370      2380      2390      2400

....|....|....|....|....|....|....|....|....|....|....|....|....|....|....|..
..|....|....|....|....|
Dmel FAS transcript protein     IMLDGAPSYVNWYTSSFKQRYTDGTNADNDNQ--SYGLAYFGIVL-
ANIDYKALVRLLIVIPTWEEKLERFAELMSNEITQPVE----------------
Lclav FAS transcript protein
TL...S.DFIKLHSQTIGKQTNQVSRDLAS.DGFQKAI.F.ARQ.NSD.SFIKAYEI.RGSKSED.T.NKMI..IG.
T-PFKS.----------------
Gleg protein FAS                .L...S.EFITLHSTLINKQVSPDNSELQT-
DGCRKS..F.IKQFNR..N.TNAYKS.QEVKDE-.IFDKMI..IGPT-SLDID----------------
Abil protein FAS                FLI..S.T..ATH.GKAS..IQP.NT.AEH----TEA.LF.MHQF-
KEV.QQKTAAE.MALK.LD.RAKLTTQIIGDACPF.K.----------------
Pmac protein FAS                V.....K.....TN....L---NTS.DQ.E--A.......M.V-
.....SLVAKV.LN....DS.VAKC..IVAA..N..TDLVSNIFGKNKKKKRST

                                       2410      2420      2430      2440
2450      2460      2470      2480

....|....|....|....|....|....|....|....|....|....|....|....|....|....|....|..
..|....|....
Dmel FAS transcript protein     ---
TIKKSATLFYKKLELADGYQPTLKLKTNVTLVKPTDNSAKLDEDYRLKEVCTKPVEVHTVEGNHRTFLIEDQSLKTI
QSILKRLFN
Lclav FAS transcript protein    ---
DL.IAGI......RA.NM.KASN.YNGPI..I.AK..FVS.NN..G.S.I.RQT.RIEELP....SI.-
SGE.V.KMATLV.T---
Gleg protein FAS                ---
DL.MAGY.L....RA.NL.R.SG.F.GP.Q.I.AN.AFIHMS...G.SQ----------------------------
---------
Abil protein FAS                ---
Q.TAA.KS..Y..KA..M.K.AS.FNG.II.A.AN..YVQGES..G.SN.SL-------------------------
---------
Pmac protein FAS
KNVM.VQA..S.....LA..K.V.SI.VSCD........E.Y...E...G.N..---N.DL----------------
------------
```

Supplementary table 1: Fat synthesis is induced plastically based on host fat content.

We analysed the data presented in Figure 2 in the main text statistically by means of a linear mixed effects model (GLMM, lme4 package) with host (lean *D. simulans* and fat *D. melanogaster*) as fixed effect, population (Japan, United Kingdom 1 and 2, Belgium 1 and 2), family and experiment (each experiment was conducted twice) as random factors, and percentage of incorporation of stable isotopes as dependent variable (n = 138). Non-significant terms (i.e. population and experiment) were sequentially removed from the model to obtain the minimal adequate model as reported in the table. When referring to "families," we are referring to the comparison of daughters of singly inseminated females, which (in these haplodiploid insects) share 75% of their genome.

Fixed effects:	Estimate	Std. error	t-value	P
Intercept	2.177	0.357	6.104	**0.001**
Host *D. simulans*	1.061	0.241	4.410	**0.001**
Random factors:	Variance	Std. error		P
Family (intercept)	0.301	0.548		**0.001**

Supplementary table 2: Fat synthesis is plastically induced in lean *D. melanogaster* hosts

Mean absolute fat amount ± 1se (in µg) was quantified in adult wasps from field-caught *L. heterotoma* populations raised on lean *D. melanogaster* hosts at two adult stages (Emerged: just after emergence; Fed: having fed for 7 days after emergence). Lean *D. melanogaster* hosts were produced by rearing the larvae on a medium containing 100 times less sugar than usual. P-values based on t-tests (with log-transformed data in case of the Belgium 2 population noted by ^) reveal whether 7 days of feeding led to a significant increase in fat content, indicating the occurrence of fat synthesis (in bold).

Population	n	Emerged	Fed	p-value
Belgium 2	31	27.40 ± 1.82	38.08 ± 3.50	**0.018 (^)**
UK 1	33	25.09 ± 2.51	40.50 ± 2.66	**<0.001**
UK 2	35	27.25 ± 2.60	38.62 ± 2.70	**0.006**
Japan	31	34.70 ± 2.72	34.36 ± 3.69	0.943

Supplementary text 1: Acetyl coenzyme A carboxylase (ACC) amino acid sequence alignment for *D. melanogaster*, *P. maculata*, *L. clavipes*, *G. legneri* and *A. bilineata* :

```
                                         10        20        30        40
50        60        70        80        90        100

....|....|....|....|....|....|....|....|....|....|....|....|....|....|....|..
..|....|....|....|....|
Dmel ACC protein transcript va  ---------------------------------------------
----------------MLITILGTL---AAFLAFLLTLIFGRGQKRSKPVQSS--
Pmac ACC protein Pmac maker-sc  ---------------------------------------------
-------------------------------------------------------
Gleg ACC protein augustus_mask
MTDQEDKSEPNLREERAAPQRKIRHRGIVEREILFPNVTESLEVAVVEDTLSILRITFEAALT.A..LA.LAC...G
VV.SRVWNASASS.GNTGYTEND
Lclav ACC protein scf718000516  ---------------------------------------------
-------------------------------------------------------
Abil ACC protein Abil maker-sc  ---------------------------------------------
----------------------------------MAKQLNR.NS..---------

                                        110       120       130       140
150       160       170       180       190       200

....|....|....|....|....|....|....|....|....|....|....|....|....|....|....|..
..|....|....|....|....|
Dmel ACC protein transcript va  ------------------AATSATTATTGDSDNGNTNHNSSV-
IAATATATTTSSKPPIAPAAPPSAVKASDKRFPKACIKKVQFSSESLRSDVDELCDQ
Pmac ACC protein Pmac maker-sc  ---------------------------------------------
-------------------------------------------------------
Gleg ACC protein augustus_mask  NSRIDRDSEQEEALNAERM.EAPVSFVV..P.---
ADP.EELE.EDSFPNE.SD.NIQMQQTIA.GLLE---R.------RRLR----------------
Lclav ACC protein scf718000516  ------------------MTET.VSFVL..P-----
DPKEELE.EDSFPE--PEANDR.QQPIL.GL.E---R.------.RLR.---------------
Abil ACC protein Abil maker-sc  -------------------------FVI..E.V.S.DN-------
--.P.DES.------.FTI..I.E-------------------------------

                                        210       220       230       240
250       260       270       280       290       300

....|....|....|....|....|....|....|....|....|....|....|....|....|....|....|..
..|....|....|....|....|
Dmel ACC protein transcript va  LKDSALSNLSNDNIRIACHQNNNN---
SSINKNQNNNSIDISISISKMSETNESNDTAAQSAEGERPSFLVGDEIDERAAEAGEACDEFPLKMQNDVRQN
Pmac ACC protein Pmac maker-sc  ---------------------------------------------
-------------------------------------------------------
Gleg ACC protein augustus_mask  ---------------------------------------------
-------------------------------------------------------
Lclav ACC protein scf718000516  ----V.AER.EK.---.AWKSSD.LLAGIVRM.SE..EDN---
EVF.REPSI.TEK..G-----------------------------------------
Abil ACC protein Abil maker-sc  ------.H.QD.D-----------------.L.G.G.GY-----
..-------------------------------------------.VW..QARLLK

                                        310       320       330       340
```

350   360   370   380   390   400

....|....|....|....|....|....|....|....|....|....|....|....|....|....|....|..
..|....|....|....|....|
Dmel ACC protein transcript va GDISERRKRLRPSMSRGTGL--
GQDRHQDRDFHIATTEEFVKRFGGTRVINKVLIANNGIAAVKCMRSIRRWAYEMFKNERAIRFVVMVTPEDLKANAE
Y
Pmac ACC protein Pmac maker-sc -------------.......--
....Y......V........K...N....R....................S....Q....V................
.
Gleg ACC protein augustus_mask -----------
....Q..VMIQA.S.L.EK..TV..P....H..............................S.........V.....
....
Lclav ACC protein scf718000516 ---------
.T....Q..VMIQA.S.QLEK..TV..P....R.....K........................S.........V...
....
Abil ACC protein Abil maker-sc PGV.-----.T....Q..VI--
M.N.LHE...TV..P....R.....K....................V...S........SV................
.

                                        410   420   430   440
450   460   470   480   490   500

....|....|....|....|....|....|....|....|....|....|....|....|....|....|....|..
..|....|....|....|....|
Dmel ACC protein transcript va
IKMADHYVPVPGGSNNNNYANVELIVDIALRTQVQAVWAGWGHASENPKLPELLHKEGLVFLGPPERAMWALGDKVA
SSIVAQTAEIPTLPWSGSDLKAQ
Pmac ACC protein Pmac maker-sc
....N...................
........D.........E...H
Gleg ACC protein augustus_mask
.....Q.......T...............I..........................NNIC.I..S..........I.
........DV........E...H
Lclav ACC protein scf718000516
.....Q...................I...I..........................NNIC.I..S..........I.
........DV........E....
Abil ACC protein Abil maker-sc
.............T...........I...V.C........................NNIA.I...DK........I.
........D..............

                                        510   520   530   540
550   560   570   580   590   600

....|....|....|....|....|....|....|....|....|....|....|....|....|....|....|..
..|....|....|....|....|
Dmel ACC protein transcript va
YSGKKIKISSELFARGCVTNVEQGLAAVNKIGFPVMIKASEGGGGKGIRRVDTTEEFPGLFRQVQAEVPGSPIFVMK
LARGARHLEVQLLADQYGNAISL
Pmac ACC protein Pmac maker-sc
..........D..N........E..............................A...SAM.......I.........
...............M.......
Gleg ACC protein augustus_mask
.............KK...ST..EC...A........V............K.ENA..L.V........I......I..
..KC...I....V..N.......
Lclav ACC protein scf718000516
.............KK...STI.EC.T.A........V............K.ENA..L.A......T.I......I..

..KC...........N.......
Abil ACC protein Abil maker-sc
..........D.......HTA...QQ.AQ....................K..NP.D.AAS.................
..KC..................I

610 620 630 640
650 660 670 680 690 700

....|....|....|....|....|....|....|....|....|....|....|....|....|....|....|..
..|....|....|....|....|
Dmel ACC protein transcript va
FGRDCSIQRRHQKIIEEAPAIVAQPEVFEDMEKAAVRLAKMVGYVSAGTVEYLYDPEGRYFFLELNPRLQVEHPCTE
MVADVNLPAAQLQIGMGIPLYRL
Pmac ACC protein Pmac maker-sc
....A..K..................
.........C.......V..F..
Gleg ACC protein augustus_mask
....................VI.K.D...E.........................TS...Y................
.IS...........A..L..HHI
Lclav ACC protein scf718000516
....................VI.K..I..E..........................S...Y................
..S...........A..L..HHI
Abil ACC protein Abil maker-sc
....................VI..QS.............................TS.H.Y................
..S...........A..LS.HKI

710 720 730 740
750 760 770 780 790 800

....|....|....|....|....|....|....|....|....|....|....|....|....|....|....|..
..|....|....|....|....|
Dmel ACC protein transcript va
KDIRLLYGESPWGSSVIDFENPPNKPRPSGHVIAARITSENPDEGFKPSSGTVQELNFRSSKNVWGYFSVAASGGLH
EFADSQFGHCFSWGENRQQAREN
Pmac ACC protein Pmac maker-sc
.........N...T.I...
....
Gleg ACC protein augustus_mask
.............D.Q...DQ.RH..Q.W...G......
...............D.H.....
Lclav ACC protein scf718000516
.............D.A...DQ.RH..Q.W...G......
...............D.......
Abil ACC protein Abil maker-sc
.........T...D.....DQ.KH..Q.W..
.................E.....

810 820 830 840
850 860 870 880 890 900

....|....|....|....|....|....|....|....|....|....|....|....|....|....|....|..
..|....|....|....|....|
Dmel ACC protein transcript va
LVIALKELSIRGDFRTTVEYLITLLETNRFLDNSIDTAWLDALIAERVQSEKPDILLGVMCGSLHIADRQITESFSS
FQTSLEKGQIQAANTLTNVVDVE
Pmac ACC protein Pmac maker-sc
....A........S.A...

```
...................I...
Gleg ACC protein augustus_mask
...........................ES.QQ.N.......L......R.D...V..A.T..A......T..AA.TG
...A........S.D.E.I....
Lclav ACC protein scf718000516
..V........................EC.QQ.C.......V......R.D...V..A.T..A......T..AA..G
...A........S.D.N..M...
Abil ACC protein Abil maker-sc
...........................KS.Q..T.......I..S..M.......M...I..A.....KT.STA.NE
..I...R....GS...NHTM...

                                          910       920       930       940
950       960       970       980       990       1000

....|....|....|....|....|....|....|....|....|....|....|....|....|....|....|..
..|....|....|....|....|
Dmel ACC protein transcript va
LINDGIRYKVQAAKSGANSYFLLMNSSFKEIEVHRLSDGGLLISLEGASYTTYMKEEVDRYRIVIGNQTCVFEKEND
PSLLRSPSAGKLINMI-------
Pmac ACC protein Pmac maker-sc
...G.N............T......N....V.........M.M.F................................
..............LL-------
Gleg ACC protein augustus_mask
.....YK..I......P.....V..G.Y..V...........L..D...V....R........I......I...D..
.............SFL-------
Lclav ACC protein scf718000516
.....YK.....T...L.T...V..N.Y..VDI.........L..D...F....R.................D.D..
..............FLVEDGGHV
Abil ACC protein Abil maker-sc
.....HK.R...T.....T...V..G......L.......I.L.VD...F....................I.D....
.T...........GFL-------

                                          1010      1020      1030      1040
1050      1060      1070      1080      1090      1100

....|....|....|....|....|....|....|....|....|....|....|....|....|....|....|..
..|....|....|....|....|
Dmel ACC protein transcript va  ------
VEDGAHVSKGQAYAEIEVMKMVMTLTSQEAGTVTFVRRPGAVLDAGSLLGHLELDDPSLVTKAQPFKGQFLQPE--
NAPVPEKLNRVHNTYKSI
Pmac ACC protein Pmac maker-sc  ------
.......N...................L.....................................Y....P...--
.P.L.........I....
Gleg ACC protein augustus_mask  ------
....G..DA............I..V..S...S.FY.K......E..T.IA.............SEYT...PPAA--
APAI.....HL.TK.RTA
Lclav ACC protein scf718000516
DAGQAS....G..DA...............V.AG...SIFY.K......E..T.IA..............EYL....
A.V--TPA......HL.AK.RAA
Abil ACC protein Abil maker-sc  ------
....G..YR.................AG...S.SY.K.A........IIAT.....A.......LYTSP.PDLDVSH
PLAS....HI..S....

                                          1110      1120      1130      1140
1150      1160      1170      1180      1190      1200

....|....|....|....|....|....|....|....|....|....|....|....|....|....|....|..
```

..|....|....|....|....|
Dmel ACC protein transcript va
LENTLAGYCLPEPFNAQRLRDIIEKFMQSLRDPSLPLLELQEVIASISGRIPISVEKKIRKLMTLYERNITSVLAQF
PSQQIASVIDSHAATLQKRADRD
Pmac ACC protein Pmac maker-sc
.....................V...
...................T...
Gleg ACC protein augustus_mask
...........D.YHLP....L.....N.................T.................S.............
......A...G.....S..S...
Lclav ACC protein scf718000516
.......F...D.YHLP...EL.....N.................T...............................
......A...G.....S......
Abil ACC protein Abil maker-sc
.....Q.F...D.Y..K...EV.....A........................PA.........S.............
..............SM....E..

1210 1220 1230 1240
1250 1260 1270 1280 1290 1300

....|....|....|....|....|....|....|....|....|....|....|....|....|....|....|..
..|....|....|....|....|
Dmel ACC protein transcript va
VFFLTTQSIVQLVQRYRNGIRGRMKAAVHELLRQYYDVESQFQYGHYDKCVGLVREHNKDDMQTVVNTIFSHSQVAK
KNLLVTLLIDHLWANEPGLTDEL
Pmac ACC protein Pmac maker-sc
N......G...............................C...H............R....................
....
Gleg ACC protein augustus_mask
.......A.................T..........T......Q.......SALI.QY...VA..TGM....N..T.
..V...M................
Lclav ACC protein scf718000516
.......T...........T.....T......K...N......Q.......SALIDQF...KTM.TS........T.
..V...M......T.........
Abil ACC protein Abil maker-sc
N...A.DG.....................D......N......L.......SAL.DKH...SM.TQI..........
..M...M......S.........

1310 1320 1330 1340
1350 1360 1370 1380 1390 1400

....|....|....|....|....|....|....|....|....|....|....|....|....|....|....|..
..|....|....|....|....|
Dmel ACC protein transcript va
ANTLSELTSLNRAEHSRVALRSRQVLIAAHQPAYELRHNQMESIFLSAVDMYGHDFHPENLQRLILSETSIFDILHD
FFYHSNRAVCNAALEVYVRRAYT
Pmac ACC protein Pmac maker-sc
....
....
Gleg ACC protein augustus_mask
SS..T.......T........A..K..............
......................V
Lclav ACC protein scf718000516
.S..T.......T........A..K......C.......
.......V..............I
Abil ACC protein Abil maker-sc
.A..N.......S........A................................E.......K..V...........

....T.................

                                        1410   1420   1430   1440
1450   1460   1470   1480   1490   1500

....|....|....|....|....|....|....|....|....|....|....|....|....|....|....|..
..|....|....|....|....|
Dmel ACC protein transcript va
SYELTCLQHLELSGGLPLVHFQFLLPTAHPNRLFSRMSSPDGLDQAAAESLGNSFVRTGAIAAFDSFEHFEMYSDEI
LDLLEDFVSPAMVNAKVLEAVEA
Pmac ACC protein Pmac maker-sc   ..D.............I...............L..A.-
EAATE.GTDN..T.Y....CM........D...........LA.ST..S........
Gleg ACC protein augustus_mask   .............EI.........NN....----------
QN.STVNH------....M...QDL.Q.SQ.A..V......LS..SS.S..I.....
Lclav ACC protein scf718000516   .............EI.......M..NN....----------
QN.SLVNH------....M...QDL.Q.NQ....V......LS..NS.S........
Abil ACC protein Abil maker-sc   ..DI........AEV..I.......PS.....VTLD.I---
.EETEPAKVFD..Q...CM...E..QQ..S.A...F..I...AN..TIS..D.NML.S

                                        1510   1520   1530   1540
1550   1560   1570   1580   1590   1600

....|....|....|....|....|....|....|....|....|....|....|....|....|....|..
..|....|....|....|....|
Dmel ACC protein transcript va ADSISDSR-HSTSINVSLSDPVTRANAAEEA-
KSTEPIHIVSVAVRETGELDDLQMAQIFGNYCQEHNEELFQRRIRRITFAALKKRQFPKFFTFRARDK
Pmac ACC protein Pmac maker-sc   ....G.G.-L..........IS........-
.......I.........M..V..........KQ.RD........................Y....N
Gleg ACC protein augustus_mask   .G--.E..-........I.-
TAEPSTTI.RGERPS..V..L.I..Q.IDNQ..TAL.RM..DW.ANNKD..IS.G...V......R..........Q
..G
Lclav ACC protein scf718000516 VG--.E..-
.........TT.EGNAQN.SGDDPA..F..L.I..IDK.NQ..AT..RV..DW.ALNKD..IA.GV..V..L....
.....L....Q..G
Abil ACC protein Abil maker-sc G---G...TN.........IDGQ.QITEDSN-
.VC.....LHIG.KDK.DE..ST.SR...SF.ER.RQD.ET.G..........HK.......Y....G

                                        1610   1620   1630   1640
1650   1660   1670   1680   1690   1700

....|....|....|....|....|....|....|....|....|....|....|....|....|....|..
..|....|....|....|....|
Dmel ACC protein transcript va
FTEDRIYRHLEPASAFHLELNRMKTYDLEALPTANQKMHLYLGKAKVSKGQEVTDYRFFIRSIIRHSDLITKEASFE
YLQNEGERVLLEAMDELEVAFSH
Pmac ACC protein Pmac maker-sc
YE..........C.YQ.........................R..........F....................
....
Gleg ACC protein augustus_mask
.V.........GC..Q......R.........S.........Q...A...Q........................D
..H...................
Lclav ACC protein scf718000516
.F...V......GC...I.....R.........S.........Q...A...Q........................D
..H...................
Abil ACC protein Abil maker-sc
.K..........C..Q......R..N......S.............AP.H.........................
....

```
                                        1710       1720       1730       1740
1750       1760       1770       1780       1790       1800

....|....|....|....|....|....|....|....|....|....|....|....|....|....|....|..
..|....|....|....|....|
Dmel ACC protein transcript va
PHAKRTDCNHIFLNFVPTVIMDPAKIEESVTKMIMRYGPRLWKLRVLQAELKMVIRQSPQSPTQAVRLCIANDSGYF
LDISMYTEQTEPETGIIKFKAYG
Pmac ACC protein Pmac maker-sc
.F...................................................L...N..A...S............
........H.DK...V...M...
Gleg ACC protein augustus_mask
.L....E........A.N......R......S.VL...........R...I..T..PA.GK..TNI..........S
I.LHL...A.D.K....R.ESFP
Lclav ACC protein scf718000516
.L......................R......S.VL...Q.......R...I..T..PA.GK..SN.....S.....S
I.LHL...AIDQK....R.ES.-
Abil ACC protein Abil maker-sc
.QSR...........I.......S....A..S.V...................T..S..T...TT...........Y
...N....VVNVD....R.E...

                                        1810       1820       1830       1840
1850       1860       1870       1880       1890       1900

....|....|....|....|....|....|....|....|....|....|....|....|....|....|....|..
..|....|....|....|....|
Dmel ACC protein transcript va  E--------
KQGSLHGHPISTPYMTKDFLQQKRFQAQSNGTTYVYDVPDMFRQMTERHWREFSKARPT-
VDIRTPDKILIECKELVLEGDN---LVEMQR
Pmac ACC protein Pmac maker-sc  .--------
.......L.............................I..........L.K.Y.M....-
....I.E......V....D...---....K.
Gleg ACC protein augustus_mask
SQNPNNPNPRI.PM..L......L...Y..A.......A.......L......QL.KT.AKYIDE.SAIEP.TM.NP
VM-DSV...V..E.---...LK.
Lclav ACC protein scf718000516  SGSANNSN-
RP.PM..L......L...Y..A.......S.......L......QV.KS.K..IDE..S-EV.TI.NPLI-
.IV....D.-D---...LK.
Abil ACC protein Abil maker-sc  T--------
...P...L......LA..Y.........QS.......Y.......VDLL.KQY.QE.MN-EVVVI.E.VM-
D.I....DPE.ESR...QK.

                                        1910       1920       1930       1940
1950       1960       1970       1980       1990       2000

....|....|....|....|....|....|....|....|....|....|....|....|....|....|....|..
..|....|....|....|....|
Dmel ACC protein transcript va
LPGENNCGMVAWRIVLATPEYPNGREIIVIANDLTYLIGSFGIKEDVLFAKASQLARQLKVPRIYISVNSGARIGLA
EEVKAMFKIAWEDPEEPDKGFKY
Pmac ACC protein Pmac maker-sc
.....T........I.......E...M........FF......P..IV.H.......SR..................
........V..............
Gleg ACC protein augustus_mask
.....DV......FT.Y...C.T..DV.L.G..I.HM.....PR..I..YR..ER.....I....FAA.........
.....L.......EM..E.....
```

```
Lclav ACC protein scf718000516
.....DV......LT.Y...C.T..D..L......H.......P..I..F...ER....GI..V.F.A......A..
.....L.R.....EA..E.....
Abil ACC protein Abil maker-sc
V.....V......LT.Y.....A..I.......I.F.M...APR..KV.GL..E...N.......AA.........
.....LY....D..N...R..R.

                                        2010      2020      2030      2040
2050      2060      2070      2080      2090      2100

....|....|....|....|....|....|....|....|....|....|....|....|....|....|....|..
..|....|....|....|....|
Dmel ACC protein transcript va  LYLSTEDYAQVANLNSVRAILIED-
EGEQRYKITDIIGKDDGLGVENLRYAGLIAGETSQAYEEIVTIAMVTCRTIGIGSYVVRLGQRVIQIDNSHIILT
Pmac ACC protein Pmac maker-sc  ...T....SR..K..........-
...P..........E....................D.....S............L.........E.......
Gleg ACC protein augustus_mask
I..TPD...RL.P....K.S....PA..S...........Y.I.....K...M......K..D.V...SI.S..A..
....L..........E.......
Lclav ACC protein scf718000516  I..TPD...RLSP....K.S....-
G..S..R...............K..........K....V...SV.S..A................E.......
Abil ACC protein Abil maker-sc  ...TP....K.SAW.....V....-
...S............F.....Q..............D....S..S..A....A.L.................

                                        2110      2120      2130      2140
2150      2160      2170      2180      2190      2200

....|....|....|....|....|....|....|....|....|....|....|....|....|....|....|..
..|....|....|....|....|
Dmel ACC protein transcript va
GYAALNKLLGRKVYASNNQLGGTQIMFNNGVTHKTEAIDLDGVYTILDWLSYIPAYIGCDLPIVLP-
NDRIERPVDFMPTKSPYDPRWMLGGRVNPVNAN
Pmac ACC protein Pmac maker-sc
......................V...Y.........L..........................E.-
....D..................A.....G..S
Gleg ACC protein augustus_mask
.NR...AV...E..........V...H....S.S.DVR.....A.A.K....V.KAK.AP...LP.LL.P...E.MY
T.........F..D...S.SDP.
Lclav ACC protein scf718000516
..R...TV...E..........I...H...IS.AI.PR.....E.V.R....M.KSK.AP...IES-
I.P.D.EIG.V...A........E.K--Q..DH
Abil ACC protein Abil maker-sc
..S........E..........I...Y....S....PR....I....K......KDKLSGV..LP.-
T.PYT.EIGY....A........A..Q..NSPA

                                        2210      2220      2230      2240
2250      2260      2270      2280      2290      2300

....|....|....|....|....|....|....|....|....|....|....|....|....|....|....|..
..|....|....|....|....|
Dmel ACC protein transcript va
DWENGFFDRDSWSEIMASWAKTVVTGRARLGGVPVGVIAVETRTVEVEMPADPANLDSEAKTLQQAGQVWYPDSSYK
TAQAIKDFGREELPLIVFANWRG
Pmac ACC protein Pmac maker-sc
E..............P.............I...........................................
.S............MI......
Gleg ACC protein augustus_mask
```

V..S.....N..Q...KP..Q...........I.C...........LHL.............IS..........A..
.........H.....FI......
Lclav ACC protein scf718000516
T..S.....G..Q...KP..Q...........I.C.I.........LHL.............VS......F...A..
........NK.....FI......
Abil ACC protein Abil maker-sc
E..A....K.......QP..Q...........I.............LK..............VS......F...A..
.....Q...K.D...FI......

2310 2320 2330 2340
2350 2360 2370 2380 2390 2400

....|....|....|....|....|....|....|....|....|....|....|....|....|....|....|..
..|....|....|....|....|
Dmel ACC protein transcript va
FSGGMKDMYEQIVKFGAYIVDGLREYKKPVLIYLPPNAELRGGAWAVLDSLINPRYMETYADPEARGGVLEPEGIVE
IKYKEKDLVKTIHRLDPTTIALK
Pmac ACC protein Pmac maker-sc
.............................I............................S..................
................A..----
Gleg ACC protein augustus_mask
............M.............TR.I.V.I...G.........V.PT...D...MF..NTS.......DA...
..F.TR.TL..M..V.HIIQK..
Lclav ACC protein scf718000516
............M.............TR.IVV.I..YG.........V.PM....H..MF..HTS............
..FRN..I...M..N.SVIHN..
Abil ACC protein Abil maker-sc
...........V............K.R..II..I...G.........V.PF..S....M.........I........
...RK...L..M..I.A.LMQ.D

2410 2420 2430 2440
2450 2460 2470 2480 2490 2500

....|....|....|....|....|....|....|....|....|....|....|....|....|....|....|..
..|....|....|....|....|
Dmel ACC protein transcript va KELDEANASGDKVRAAQ-------------------
VDEKIKARIAVLMHVYHTVAVHFADLHDTPERMLEKECISEIVPWRDSRRWLYWRLRRLLLEDA
Pmac ACC protein Pmac maker-sc ---

Gleg ACC protein augustus_mask EQ.SNTSSP-------EERSQ---------------
IEA...E.EQQ.EPM.RQI................INA.ND.....R..KL.......R.F.EE
Lclav ACC protein scf718000516 EK.ASCGSA-------EERAS---------------
.ERE.HD.ECH.ESM..Q..................NT.Q...A..SA..I.......R....R
Abil ACC protein Abil maker-sc
EK.KML..ANVPIEILERRGSVTQTPERKKTPEIIA.EKE.VE.ENY.LPM..Q...N...........H..GT.LD
.....K..TI..........Q.R

2510 2520 2530 2540
2550 2560 2570 2580 2590 2600

....|....|....|....|....|....|....|....|....|....|....|....|....|....|....|..
..|....|....|....|....|
Dmel ACC protein transcript va
YIKKILRAQDNLSVGQAKQMLRRWLVEEKGATEAYLWDKNEEMVSWYEEQINAE---
SIVSRNVNSVRRDAIISTISKMLEDCPDVALDAVVGLCQGLTP
Pmac ACC protein Pmac maker-sc ---
--------------------------------------K..SF------------

```
Gleg ACC protein augustus_mask
IRSEV.ST.PG.DIR.VGA.....FI.D..T..S....QD.TAAR.L.N.L.D.--
N.V....IAC.KK.TVVTR.KES..AY.E.R.N.MLEIVHR.HS
Lclav ACC protein scf718000516
IRSE.IST.PG.D.R.VDA.....F..D.....S....QD.VVAT.L.A.CEN.--
S.V.M..ISC.KN.S.VTRVKEA..V..E.RF...LEIVNR.Q.
Abil ACC protein Abil maker-sc
V.TQL.ETNS..GI..GEA.....F......S.G.K..N..AV.E.L.K.MSV.NEN.ML...LHA.KK..L.QK.K
NSI.....L......EIL.K.ND

                                       2610      2620
                                ....|....|....|....|....|...
Dmel ACC protein transcript va  VNRGVVVRTLAQMQLNEETSNSNQG---
Pmac ACC protein Pmac maker-sc  -----.QY.CF-----------------
Gleg ACC protein augustus_mask  TE.AELL...S.IEASGQEHHNDSNVSS
Lclav ACC protein scf718000516  AEIAELQ.....LESTSQENHNDSSASS
Abil ACC protein Abil maker-sc  NQKAE.I...S.V.PET.S---------
```

Supplementary text 2: Fatty acid synthase (FAS) amino acid sequence alignment for *D. melanogaster*, *P. maculata*, *L. clavipes*, *G. legneri* and *A. bilineata*:

```
                                                10        20        30        40
50        60        70        80        90        100

....|....|....|....|....|....|....|....|....|....|....|....|....|....|....|..
..|....|....|....|....|
Dmel FAS transcript protein    MPARF-------
AEEVITAEPAQRAAPQLDLGGGHYVPRQQHLNDEIAITGFSGRLPESSTIEEFKQNLFDGVDMVNDDPRRWERGLYG
LPDRIGKLKDS
Lclav FAS transcript protein   .....ESVNAPIVRDQ.IPNGT-----------------
TYSID.D.V............N......K....I.L.T..E...PS..H...T.T....--
Gleg protein FAS               ...Q.ESMNTPIVR.PVVTNGSR----------------
SFSMD.D.V.S.........EN....QK...E.I.L.T..E...PS..H...T.T....--
Abil protein FAS               ....-----VPEMR-----NGE-----------------
CHQA...VV...L.......Q.......Q......L.T..E...TA..H...T.T..I.--
Pmac protein FAS               .....-------VDG..NENGGRN.PHD.QERF.LHSSGPMTVS.-
..............N.....K...E.............K........L..I..E

                                               110       120       130       140
150       160       170       180        190       200

....|....|....|....|....|....|....|....|....|....|....|....|....|....|....|..
..|....|....|....|....|
Dmel FAS transcript protein
DLENFDQQFFGVHQKQAECMDPLLRMLLELTHEAIIDAGLNPSDLRGSRTGVYIGVSNSETEQHWCSDADRVNGYGL
TGCARAMFANRISFTFDFKGPSY
Lclav FAS transcript protein
.IAS..AT.....A...HV...Q................I...EI...K...F....D..SDEF.TA.P.M......
...C....P....Y....T...F
Gleg protein FAS
.IAS..AT.....A...NV...Q..LM..A.....V...F..T.V..T....F....S..SDDF.TRSPET......
...C....P....Y....N....
Abil protein FAS
.ITH..AT.....A...HL...Q...........L....I...EF...N...FV...E..SSEQ.TR.P.AI...S.
...C....P....Y...L.....
Pmac protein FAS
...H......S...........QM.....C.Y.....S.V...EV............A.....Y.TA.Q........
...........V.Y.........

                                               210       220       230       240
250       260       270       280        290       300

....|....|....|....|....|....|....|....|....|....|....|....|....|....|....|..
..|....|....|....|....|
Dmel FAS transcript protein
SIDTACSSSLYALEQAFSDMREGKVDNALVAGAGLILKPTMSLQFKRLNMLSPDGSCKAFDESGNGYVRSDGCVVLL
LQRTSAARRVYASILNVRTNTDG
Lclav FAS transcript protein
AV.......M..MH..VAA..T.QC.S.I.G.VN.V....S....H......M..A.....A........EAA..IF
..KARNS.....TVV.SK.....
Gleg protein FAS
A.........F.MH..IAA..S.EC.A.I.G.VN.C....A....HK.S...ME.A.....A........EAV..VF
..KSKD......TVV.SK..V..
```

Abil protein FAS
A........MF..Q..YAAIKS.QC.S.I.G.VN.L....N....H..G...AE.K.....A..S....AEAA..IV
..KA........T..GAK.....
Pmac protein FAS
.V.......F...........D..C.S.V...L.V....................M...................IF
..K..QSK....TV.........

310 320 330 340
350 360 370 380 390 400

....|....|....|....|....|....|....|....|....|....|....|....|....|....|....|..
..|....|....|....|....|
Dmel FAS transcript protein
FKEQGITYPIGKMQNRLIRETYEEIGLNPADVVYVEAHGTGTKVGDPQEVNSITDFFCKDRTTPLLIGSVKSNMGHS
EPASGVCSVAKILIAMEEGVIPG
Lclav FAS transcript protein
N.VE...F.S.A...K.M..V.A.V.VD.V.......................A.L.....KK......I.......
.....L..I..M.L...A.....
Gleg protein FAS
Y.DL.....S.S...K.M..I.D.C.V..S..T....................A.L.....KN...L..........
.......AI..L...L.T.M..P
Abil protein FAS
S......F.S.QV.....N.V.ADS.IE.N.......................A.....N.K....L..........
.....L..I..M.....S.Q..A
Pmac protein FAS
Y........D.R.............N.D.NE.A..........................N.................
........I..V..........A

410 420 430 440
450 460 470 480 490 500

....|....|....|....|....|....|....|....|....|....|....|....|....|....|....|..
..|....|....|....|....|
Dmel FAS transcript protein
NLHYNKPNPDLYGLVDGRLKVVDRNLPWNGGIIGLNSFGFGGANAHVILKSNPKPKALTPK--
DGALKVVLASGRTFEAVEQLLESASTNADDDEYLQLI
Lclav FAS transcript protein
...FKS..K.IPA.S....Q....SM.....LVAI...........LV.R......IA-
.VLDVNVP.I.PV....DD..NLF.DRIKEHEK...FTSMV
Gleg protein FAS
...FQN..K.IPA.S...IQ..TQPTAYK.NLMAV...........ILVRGHS...LS-
.VMDR.VP.L.AV....N...NVM.DKIKEHHR...FIA..
Abil protein FAS
...FKN..T.IPA.C...I...AT.EK.T...V.V..............R......ENW.V--
EQLPRL.VV....ED..NHF.DKIKEQSH.E.FYAML
Pmac protein FAS
....KN........M........K....E.....I.....................TI..T--
V.PP.M.VC.....D..QE...D.TSHR......A..

510 520 530 540
550 560 570 580 590 600

....|....|....|....|....|....|....|....|....|....|....|....|....|....|....|..
..|....|....|....|....|
Dmel FAS transcript protein
NEIHSKAIPNHFFRGYGVVSSKGTHQREVIESNDDKRPIWYIYSGMGSQWASMAKDLMKIEAFAKTIQRCADVLKPE
GVDLIDVLTRSTDKSFENILNSF

Lclav FAS transcript protein QDL.ANN.TG.GY..FQILGDVN.--
..IDQVGSE.......F.......SG.GRA.FC.DT.QSA.R...EA.....I...NLILNG.EE..Q.VV...
Gleg protein FAS H...N.N..G.N....QILGGED.--
..IL.NHSA.....FVF.......PG.G.E.LHLDV.NRSLR...EA.RS.....M.IIQNG.NET....I...
Abil protein FAS .N..A.N.TG.NY..FA.LGDNEI--
.D.SMVGNE.K...FVF.......PG..R.....DL.QQ..K.A.QA.N.Y....E.I.LN..EETLT.VR...
Pmac protein FAS
.D....N..L.YY...C.MDT..SL....L.F..EN..V...................QF.V..NS.H...KA.R..
.I..V........L..D......

610 620 630 640
650 660 670 680 690 700

....|....|....|....|....|....|....|....|....|....|....|....|....|....|....|....|..
..|....|....|....|....|

Dmel FAS transcript protein
ISIAAMQVALTDLLSSLGIHPDGIVGHSVGELGCAYADGCFTPEQTVLAAYWRGKSILDTQLAKGKMAAVGLSWEDA
HSRVPSDCFPVCHNSEDNCTISG
Lclav FAS transcript protein
V....I.IG.V.V.NLI..Q....I...I.....G....TM..S...........T...GN.PP.A.........E.
QK.C.PEIVLA...AA.SV....
Gleg protein FAS
V....I....V...T........................T......I....L...A.AESD.PA.C..........T
KA.C.P.VV.A...AA.SV....
Abil protein FAS
....SI.IG.LEI.K..N.E....L...I..V.......TL.L...IQL.WA..TA..ESD.PP.A......T..EC
KK.C...I.........SV....
Pmac protein FAS
..............T..N.K.....................S...............Q..K.PP....SI..D..E.
.K.M.A.........A.......

710 720 730 740
750 760 770 780 790 800

....|....|....|....|....|....|....|....|....|....|....|....|....|....|....|....|..
..|....|....|....|....|

Dmel FAS transcript protein
PEASIEALVAKLNAEGVFAKAVNSSGYAFHSKYIAEAGPKLRKSLEKIIPNAKNRTARWISTSIPESAWNTPVAKQS
SAAYHVNNLLSPVLFHEALQHVP
Lclav FAS transcript protein
.PEPLAKF.EE.KSQEI...Q.H...C........SV.....TI.......P.Q.SS....S....T....SL.QL.
.P..Y..........Q...A...
Gleg protein FAS
.TG.V.KFIEE.KK.EI.....K.N.I........S......AG....LT.P.Q.SS....S.......G..L.QL.
.P.............Q...A...
Abil protein FAS
.P...DKFT.E.TK..I...K.....F........A.......A.DT..Q.P.A.S.............G..L.Q..
N............Y....TK.I.
Pmac protein FAS
.....D....Q.SS........K............D..........R.........NK..............I....
...............Y.....I.

810 820 830 840
850 860 870 880 890 900

....|....|....|....|....|....|....|....|....|....|....|....|....|....|....|....|..
..|....|....|....|....|

Dmel FAS transcript protein
KNAISVEIAPHGLLQAILKRALGPDATNLSLVKRGHENNVEFFLTNVGKLFAAGAQPQVLTLVRPISYPVGRGTPML
NSKVGWDHTQKWLVAKF-GKETS
Lclav FAS transcript protein
D...VI.....C......R.SFPSTV..IG.H..D.SD.LA.L...I...YV.....ILSK.YP.VT..........
..MIL...STQ.S..D.S..SGN
Gleg protein FAS
ED..AI.....C......R.S.PKTV..I..H..D.TD.MNYL.S.....YC......ISK.YP..NF........I
..MIK...SVQ.G..NYAQNSAR
Abil protein FAS
D...VI..............G.SK.CA.I........D.AR.L.S.I.RI.N..G..NIAN.YH.V.F.........
A.MIE...STE.S..NYCD.NDR
Pmac protein FAS
....AI....T.................V.............L.M.I...Y....K.....MFK.............
........S...N.P.Y-....T

910 920 930 940
950 960 970 980 990 1000

....|....|....|....|....|....|....|....|....|....|....|....|....|....|....|..
..|....|....|....|....|
Dmel FAS transcript protein
SGETIVEVDLSKEDDAFLAGHTIDGRILFPATGYMTLAWQTFAKMQGSEFHKTPVVMENLVFHRATILNKNAVVKFG
INFFDGTGAFEICESGSLAVSGK
Lclav FAS transcript protein
..QSV..F.....S.SYI...C............L.IV.K....LR..SYE.....F.DVQ.L....MP.EGS...I
..I.E.......S....ST.I..
Gleg protein FAS
...S...I...T.S..Y.................L.IV......LHNE..NRM.IIL..VQ......MP.EGK...L
..I.E...D........I..T..
Abil protein FAS
..QFVIDI.....EHKY.V...............L..V.K.....RNQD.EQL..II.DVK.M....MP.EGS...L
..I.E.S.E........V.....
Pmac protein FAS
....VI.IN.G..E.S.F.....................M.....K.M.YQ.C..I...I..........EG.....
L.......N.....G........

1010 1020 1030 1040
1050 1060 1070 1080 1090 1100

....|....|....|....|....|....|....|....|....|....|....|....|....|....|....|..
..|....|....|....|....|
Dmel FAS transcript protein ITIPESIDNEELPLEEQTPSAVAKE-----
LGTNDVYKELRLRGYDYGGIFRGIVRSDTVASTGKLQWVDNWISFMDTMLQFSILSKNLRELYLPTRIER
Lclav FAS transcript protein .R.S.D.EKDQ.N.---
PIPVTCN.PDLLE.K......D........S...Q..KS..NR.I..N.A.NND...Y.......A..G..T.D.F.
...LQY
Gleg protein FAS .RRA.Y.E..Q.N.---
PIPVLR..ENILD.N...I..D........S...Q..KSA.NRGII...T.SND..............G..T.D.F.
...LQY
Abil protein FAS .YVA.EPEKQF.T.---
PKHTLI..KDILD.N.P.I...........D......HSA.NYGLV...K.EQ.....I......N.....T.....
...LQ.
Pmac protein FAS .S...D.EM.....DALPA.TLG..-----
.N...............S.......K.....N..Q...AE..V..........................K

1110 1120 1130 1140

1150 1160 1170 1180 1190 1200

....|....|....|....|....|....|....|....|....|....|....|....|....|....|....|..
..|....|....|....|....|
Dmel FAS transcript protein
AVINPAKHFELLSALTKEEQVETGLPVQWYSDINVIKSAGVELRGLKANLAQRRPGTQAPPTLERYQFVPNINTTDL
NENSEKARLHALDVAIQVIIENS
Lclav FAS transcript protein .A...ER.IQ.VEK.QEN.----
NI..FH..NVGIV..G......M.SSI.P..QQ...D.K..K.S.I.YE..QA.V.DP..SK....TSLL..VR..I
Gleg protein FAS .A....L.MH.V.G.KSD.----
....YS..N.GIL..G.I....M..S..P..QQA....KH...T...YETNNA.V.DPQ..KV..MC.LF.I.C..M
Abil protein FAS .I...VE.IR-----NAK.----
HVT.SM.R..D....G.........S..P..QQS.SA....Q...L.YL.MNQVVDEQI-----T.T..S.IAL...
Pmac protein FAS
........LATV.K.SE.YLTLN....YM.G.......G...M.....S..SK...S.N.......T.L..V.YAE.
H.....S..Q..T..L.T.M...

1210 1220 1230 1240
1250 1260 1270 1280 1290 1300

....|....|....|....|....|....|....|....|....|....|....|....|....|....|....|..
..|....|....|....|....|
Dmel FAS transcript protein
SGAVKLKGVELANGRNPDVLVANRLLQIIEGEPVLTGDVAVVTSNNNEETITAALG-
DSGVRVVSKDVLKEPVEQNCHFVFGIDVLSRPDTKTLENSIAS
Lclav FAS transcript protein G.-I.I.AI.TTME...EA.LTPIV.D.LLS..M.AV.LKLA.T--
VPDNY.PIME-QCN.KTTVV.IHSS..G.DMQLIITA.IMNNQMIAAVK.LE..
Gleg protein FAS GS-M...II.V.GE.SAES.L.PTVMDVLYS..LMSV.IQIA.T--
TP..YN..ME-QYN.KT.VR..NSN.AG.DL.T.IAP....NKNVNM.K.IA..
Abil protein FAS G..L.M.V...QGSK.IEQ.LIPKVQG.L.CQ.M..VE-SILV.--
Q.NID....E-EKSIK.SR..PSADAF...A...LMS...AYNKSEV.T.AFK.
Pmac protein FAS
Q..I.I..............M.VK.........TI.A....A.Y.......AST..T.....II..NI.E.......
..LYAL........MI....K.T

1310 1320 1330 1340
1350 1360 1370 1380 1390 1400

....|....|....|....|....|....|....|....|....|....|....|....|....|....|....|..
..|....|....|....|....|
Dmel FAS transcript protein
IRENGFLILEETLPTYTKTGRALLTKFGFVAVQEQSLGATRVLVLARKAVDLKTRKSVVVVATEQNFNWVDDLKAAL
ATAATEEQYVYVVCQGEELFGAV
Lclav FAS transcript protein .KPG..ILT..ATEI----DESI.KGSSLIVIGK.VVPG-
KSYI.LK.K-EEMD-VPL.IKV..K..S..N...V..KKSE..G.K.L..S....AL.L.
Gleg protein FAS LKNG..A.....GAV----DMK..NGT.LLYAGK.ISAG-
KTYI.LK.R-ED.K-EPIIIQI..R..S.LEGV....KKSE..G.E.LL.S.....L.L.
Abil protein FAS LKPG..VLF..SSNF---SDYS.F.SQELEI.YQ.RTPM-
KIYI....Q.QVAQ-DAIIIEV..NTYS..EPI.Q.MKESE.NNRKI.LIV.....S.L.
Pmac protein FAS
.KD.....F..STTS.G.SS.D..H.Y.LIV.T..VI.GS....M...P....Q.DA...HV..A..D.LE...E..
.K..EI.R...............

1410 1420 1430 1440
1450 1460 1470 1480 1490 1500

....|....|....|....|....|....|....|....|....|....|....|....|....|....|....|..

..|....|....|....|....|

Dmel FAS transcript protein

GLMTCIKNENGGKLARLVFVQDAKAEKFSLTSTLYRQQLEKDLISNVLKNGAWGTFRHLKLETQQ--

ATLQVEHAYVNALVKGDLASLKWIEAAQADTAA

Lclav FAS transcript protein

.....VRQ.A..MNV.YF.I..VN.SA...DDAF.AK.FD.QVMA....G.Q..SY...R.DK.SDIPS.......I

...TR...S..R...GPLCYYEP

Gleg protein FAS

.F....RR.P..MN..Y..I..KN.P..G..TPF.AD..S.Q.A.....G.Q..SY...R.DQ.NDASS.......I

.T..R...S..R...GPLSYYQP

Abil protein FAS

.MVN.L.Q.P..VNM.A.LI..T...T.N.S.KFFVD..Q...VH......I..N....S.SMEK--

S........I.T.TR..........GPLGYYNN

Pmac protein FAS

.F.N............M..I..KN.......NK..AE..S....N.....SV...........DV--

.............T............P.L-CS.

1510 1520 1530 1540

1550 1560 1570 1580 1590 1600

....|....|....|....|....|....|....|....|....|....|....|....|....|....|....|..

..|....|....|....|....|

Dmel FAS transcript protein

TVDKNLETCTVYYAPINFRDVMLTSGKLAADALPGDLAEQDCVLGLEFAGRDTQGRRVMAMVPAKSLATTCVASKRM

MWQIPEKWTMEEASTVPCVYSTV

Lclav FAS transcript protein

EKFVGQ.F.D.....L....I..AT...PP........S...I.........SS.....G..A.RG....LL.DPGF

..EV.D...L...A.I.V..A.S

Gleg protein FAS

EKYP.T.M.S.....L....I..AT...PP........G...I.....S....K....IG.IA.RG....VL.DPGF

L.EV.D...L...A.I.V..A.S

Abil protein FAS DD-

P.A.L.S.....L....I..AT...PP........G...I.....S...SR.....G..A.......VL.DPGFL.E

V....SL...A.I.V..G.S

Pmac protein FAS

LL.Q...L...............S....S.........Q.............SK...I.................N.

..E..DN.............A..

1610 1620 1630 1640

1650 1660 1670 1680 1690 1700

....|....|....|....|....|....|....|....|....|....|....|....|....|....|....|..

..|....|....|....|....|

Dmel FAS transcript protein

YYALVVRGQMKKGEKILIHAGSGGVGQAAISVALAHGLTVFTTVGSKEKREFLLKRFPKLQERNIGNSRDTSFEQLV

LRETKGRGVDLVLNSLSEEKLQA

Lclav FAS transcript protein

........RLRP..S.............S.AI..HA.C......S........K.T..Q.TDK..............

.T..G..................

Gleg protein FAS

....F...SLRP..S......T......S.AI..H..CK..............K.T..Q.TD...............

.T..N.....V.....A......

Abil protein FAS

....I...GLRP..S..V...T......S.AI..HM.CK.....S.QA..D..K....Q.TDNQ............I

.TQ.............AG.Q...

Pmac protein FAS

...........................S...I..H..................K....Q.KDSH......C....MI

M.Q.Q...........A......

1710 1720 1730 1740
1750 1760 1770 1780 1790 1800

....|....|....|....|....|....|....|....|....|....|....|....|....|....|....|..
..|....|....|....|....|

Dmel FAS transcript protein
SIRCLGLNGRFLEIGKFDLSNNSPLGMSVFLKNTSFHGILLDSVMEGEEEMQNQVVSLVAEGIKTGAVVPLPTSVFN
DQQVEQAFRFMASGKHIGKVVIK
Lclav FAS transcript protein
.V...AKD...............A....F.............ALFDTNGPEKKE..R..Y....S...R...AT..T
E..I..G.....A.......LL.
Gleg protein FAS
.V...ANG..............N...........T.......ALFDTDCPEKRE..KI.N....N...R...STI..
EN.I..G..Y..T.......LL.
Abil protein FAS
.V...ANG...C....V......A....L.....T.......ALF.SDCSEKKE.MR..S...AN...Q...ST.YG
ET.A................LL.
Pmac protein FAS
.V...D..............S...R........
EH...S...............V.

1810 1820 1830 1840
1850 1860 1870 1880 1890 1900

....|....|....|....|....|....|....|....|....|....|....|....|....|....|....|..
..|....|....|....|....|

Dmel FAS transcript protein
VRDEEAGKKALQPKPRLINAIPRTYMHPEKSYILVGGLGGFGLELTNWLVTRGARYIVLTSR-------
SGVKTGYQGLMIRRWQERGVKVVIDTSDVTT
Lclav FAS transcript protein I.....-
Q.ITR.AMKTVA.......N.D...V.........M..A..MI....KN......XXXXXXXX.IR....A.C....
R.M.ITIQ.S.C....
Gleg protein FAS I....P-
N.IVPYS.KTVP.......N.....V.........M..A..MI....K..I....-------
..IR....S.C....M.M..NIHVC.H....
Abil protein FAS I....S-
R.T.I.AIKTVT...K...DT..V..................I....KK......-------
..I.....SMC....RSQ..T.L.S.A.A.K
Pmac protein FAS
.....D.R.T.K.SS..V...............I................S...K.L..S..-------
..I.....S.............L.......S

1910 1920 1930 1940
1950 1960 1970 1980 1990 2000

....|....|....|....|....|....|....|....|....|....|....|....|....|....|....|..
..|....|....|....|....|

Dmel FAS transcript protein
AAGAKKLLENSNKLALVGGIFNLAAVLRDALIEDQTAKDFKTVADPKVTATKYLDQFSRDICTELDYFICFSSVSCG
RGNIGQTNYGLANSAMERICEQR
Lclav FAS transcript protein
EV..DS..KEA....P...............M.NLEEDH..V.TL...NG.RN..AS.KKF.P...F.V........
...M...............M...
Gleg protein FAS
LS..EE..KVC.RI.P.................NLDEGQ..A.VA..I.G..N..TA..SL.PS....VV.......

...M..............V.S.
Abil protein FAS
PE..RQ..NE.A..GPI.A...........FM.NLSEA..N..CK...D......AA...LAAN..H.V....I...
...A..S.......V.....M.
Pmac protein FAS
DK.CQQ..I.A..F.................L....V...Q..CES..QG......Y..AM.S.....V.......
.....................L.

2010 2020 2030 2040
2050 2060 2070 2080 2090 2100

....|....|....|....|....|....|....|....|....|....|....|....|....|....|....|..
..|....|....|....|....|
Dmel FAS transcript protein
QVSGFPGTAIQWGAIGDTGLVLENLGDNDTVIGGTLPQRMPSCLQTIDLFLQQPHPVVASMVVAEKRKSD-
QSAGVSLIATIANILGLRDTKNIQDGASL
Lclav FAS transcript protein
.AV.L..L.........V..I..TM.N.E.EV........A...M.M.S.........L....L..RQ.AGDS.SQ.N
.LDAVG....IK.V.TVNMNN..
Gleg protein FAS
.GI.L..L.........V..I.DTM.N...EV........W...S.M.T.........L....L...N.PTDSANQI.
.VDAV.....IK.....NVNN..
Abil protein FAS
.SV.L..L.........V..I..TM.G...EV......KIS..MA.M.I.......A......L......G-
GDNQ.K.TDAV.....IK...TVPAI...
Pmac protein FAS
.A.....L..............I.....N...........T.......F.........L............-
..G.....SC...............S...

2110 2120 2130 2140
2150 2160 2170 2180 2190 2200

....|....|....|....|....|....|....|....|....|....|....|....|....|....|....|..
..|....|....|....|....|
Dmel FAS transcript protein
ADLGMDSLMSAEIKQTLERNFDIVLSAQEIRQLTFGALKAMDGGADVKP--
AAAAPAAAAGVPEANITSGGSSRTASPMGDGTQVVFT-TSLIPTEAIVQ
Lclav FAS transcript protein
.........GT.........Y.L.........A....K.LELSS.SAEANEV.SQS..NSS--------------
LTETDP.EFLFQCSG.EIV.PKSLI.
Gleg protein FAS .........GT........G..L...P....N....K.MELSSD--
-----VSI..STS------------------ESQPENLLFQYSSNEIV.F.PL.K
Abil protein FAS .........G..........Y.L..N.....A...AR.VELES.G-
-----.ST....S-------------------DN..KNL.Q.V-DE.M..QLL.N
Pmac protein FAS
.........G..............M.P.........IQ..QLS...ESSD--...S..SPV----------
RR.PSP..F.....M..S-.E.M..Q...R

2210 2220 2230 2240
2250 2260 2270 2280 2290 2300

....|....|....|....|....|....|....|....|....|....|....|....|....|....|....|..
..|....|....|....|....|
Dmel FAS transcript protein
LDTKAPANSKQSPIFFISPIEGFASALEPLAKRLEVPAYGLQYTEAVPSDSLESAAKFFIKQLRTVQPKGPYKLAGY
SFGCLLTYVMAGILEETNEVANV
Lclav FAS transcript protein .---

```
QSTSE.GE...V.HA...VV.S.KS..SE..R.VW...C.KDA.L..IPNL.TYY.QEMK..KKQ...SII.....A
CVAFE..LQ..KAG.T.EL
Gleg protein FAS                F---
DSIGIGKP...MVHA...SVAG.KL..SA.NITVF.I.C..D..L..IPEL.AHYV.LMTS..KV...R.F.....A
CVAFE..LQM.AIGHKLDL
Abil protein FAS
MNNVDSTE..KT...ILH....AVTI.KKF.QEIQA.V..I.C...A.LS.INDL..YY.E.IK.M......T.I..
...ACVGFE.GIQ..AM..KVKL
Pmac protein FAS
.QSA...E..KR.L.VV.......D..KQ..S..DC.V....C.AEANLE.IDTL.D.YL..I....AR...AI...
.Y.A.VG..IVLH..KMK.N.RL

                                       2310      2320      2330      2340
2350      2360      2370      2380      2390      2400

....|....|....|....|....|....|....|....|....|....|....|....|....|....|....|..
..|....|....|....|....|
Dmel FAS transcript protein     IMLDGAPSYVNWYTSSFKQRYTDGTNADNDNQ--SYGLAYFGIVL-
ANIDYKALVRLLIVIPTWEEKLERFAELMSNEITQPVE----------------
Lclav FAS transcript protein
TL...S.DFIKLHSQTIGKQTNQVSRDLAS.DGFQKAI.F.ARQ.NSD.SFIKAYEI.RGSKSED.T.NKMI..IG.
T-PFKS.----------------
Gleg protein FAS                .L...S.EFITLHSTLINKQVSPDNSELQT-
DGCRKS..F.IKQFNR..N.TNAYKS.QEVKDE-.IFDKMI..IGPT-SLDID----------------
Abil protein FAS                FLI..S.T..ATH.GKAS..IQP.NT.AEH----TEA.LF.MHQF-
KEV.QQKTAAE.MALK.LD.RAKLTTQIIGDACPF.K.----------------
Pmac protein FAS                V.....K......TN....L---NTS.DQ.E--A.......M.V-
.....SLVAKV.LN....DS.VAKC..IVAA..N..TDLVSNIFGKNKKKKRST

                                       2410      2420      2430      2440
2450      2460      2470      2480

....|....|....|....|....|....|....|....|....|....|....|....|....|....|....|..
..|....|....
Dmel FAS transcript protein     ---
TIKKSATLFYKKLELADGYQPTLKLKTNVTLVKPTDNSAKLDEDYRLKEVCTKPVEVHTVEGNHRTFLIEDQSLKTI
QSILKRLFN
Lclav FAS transcript protein    ---
DL.IAGI......RA.NM.KASN.YNGPI..I.AK..FVS.NN..G.S.I.RQT.RIEELP....SI.-
SGE.V.KMATLV.T---
Gleg protein FAS                ---
DL.MAGY.L....RA.NL.R.SG.F.GP.Q.I.AN.AFIHMS...G.SQ----------------------------
---------
Abil protein FAS                ---
Q.TAA.KS..Y..KA..M.K.AS.FNG.II.A.AN..YVQGES..G.SN.SL-------------------------
---------
Pmac protein FAS
KNVM.VQA..S.....LA..K.V.SI.VSCD.......E.Y...E...G.N..---N.DL-----------------
------------
```
